# The Prevalence and Associated Factors of Cancer‐Related Worries in Adult Survivors of Childhood Cancer: A Systematic Review

**DOI:** 10.1002/pon.70101

**Published:** 2025-02-13

**Authors:** Anne Maas, Anne Westerweel, Heleen Maurice‐Stam, Leontien C. M. Kremer, Alied M. van der Aa‐van Delden, Daniël Zwerus, Elvira C. van Dalen, Martha A. Grootenhuis

**Affiliations:** ^1^ Princess Máxima Center for Pediatric Oncology Utrecht The Netherlands; ^2^ Department of Pediatrics Amsterdam UMC University of Amsterdam Amsterdam The Netherlands; ^3^ University Medical Center Utrecht Utrecht The Netherlands; ^4^ Wilhelmina Children's Hospital Utrecht The Netherlands

**Keywords:** cancer, cancer‐related worries, childhood cancer survivors, fear of cancer recurrence, financial worries, health‐related worries, interpersonal worries, oncology, psycho‐oncology, systematic review

## Abstract

**Background:**

Many childhood cancer survivors (CCS) experience cancer‐related worries (CRW), for example about late effects and cancer recurrence. CRW are associated with lower quality of life (QoL) and maladaptive health care use. We examined the prevalence, severity, and factors associated with CRW in adult CCS.

**Methods:**

We included quantitative studies of ≥ 100 participants reporting on prevalence, severity, mean scores and/or associated factors of CRW among CCS aged ≥ 18 years, diagnosed at ≤ 21 years, and ≥ 2 years post‐diagnosis. We searched MEDLINE/PubMed and APA PsycINFO, hand‐searched reference lists, and consulted experts. Risk of bias was assessed using the Cochrane Childhood Cancer Risk of Bias Criteria. Results were synthesized descriptively.

**Results:**

The 17 included studies with a total of 26,306 CCS identified three main themes of CRW: health‐related, financial, and interpersonal. Most prevalent were health‐related worries regarding future health (88%–92%), late effects (83%), cancer recurrence (25%–88%), second cancers (50%–91%), and infertility (34%–68%). Factors associated with increased CRW varied depending on the specific CRW. These included female sex, more pain, anxiety, depression, chronic conditions (e.g., neurologic, being overweight), and treatment history (chemotherapy, radiotherapy).

**Discussion and implications:**

Although most included studies used single items to assess CRW, this review underscores that health‐related worries are particularly prevalent among CCS. Effectively identifying CCS at high risk, for example through using validated measures, and addressing severe CRW can facilitate adaptive healthcare use and improve QoL among CCS. Potential interventions can be providing information about late effects, psycho‐education, discussions during follow‐up care, and targeted psychosocial support for those with severe CRW.

## 1 Introduction

Due to improvements in diagnosing and treating childhood cancer, the number of childhood cancer survivors (CCS) reaching adulthood is rapidly increasing [1]. Survival comes at a cost for many CCS, including the risk of late effects like organ dysfunction and secondary cancers, resulting from their primary cancer and its treatment [2, 3]. Although systematic reviews and large cohort studies generally show that CCS function well psychosocially based on generic psychosocial measures [4, 5, 6], several large studies have demonstrated that CCS report persistent survivor‐specific challenges, such as worries about cancer‐related issues, even years beyond their diagnosis [7, 8, 9].

While there is no agreed‐upon definition of cancer‐related worry (CRW), in this systematic review it is defined as any worry, concern or fear related to childhood cancer, its treatment, and its aftermath, including late effects. Studies have documented a wide variety of CRW among CCS, including fear of cancer recurrence, and worries about late effects and infertility [7, 8, 9]. Up to 88% of CCS have reported worries about their health [10].

Awareness of cancer‐related risks can promote vigilance toward unusual physical symptoms and enhance adherence to health screenings [10]. However, excessive CRW can disrupt daily functioning and diminish quality of life [11]. In CCS, CRW are associated with anxiety and depression [8], sadness and suicidality [12], and substance use [13]. Additionally, CRW can lead to excessive reassurance‐seeking, which can in turn increase healthcare costs, while in other CCS it can lead to avoidance of medical examinations [14, 15]. Longitudinal data indicate that the prevalence of several CRW is largely stable over time [10, 16, 17]. Therefore, addressing CRW through targeted psychosocial care is crucial.

Despite the importance of CRW, no systematic review has yet specifically addressed this topic among CCS. In contrast, CRW, particularly fear of cancer recurrence, has been extensively studied in adult‐onset cancer survivors. Systematic reviews in this population indicate that a significant proportion experience fear of recurrence, especially those who are younger, female, and experiencing physical symptoms such as pain [18, 19].

The present systematic review offers insight into the prevalence, severity, and factors associated with CRW in adult CCS. Understanding these aspects is essential for identifying CCS with severe CRW impacting daily life and potentially requiring clinical attention, and for developing effective psychosocial interventions.

## 2 Methods

The methodology is based on the Preferred Reporting Items for Systematic Reviews and Meta‐Analyses (PRISMA) guidelines [20].

### 2.1 Eligibility Criteria

We included quantitative studies reporting on CRW prevalence, severity, mean scores, and associated factors in adult CCS, published in English. Studies with ≥ 100 participants assessing self‐reported CRW through quantitative questionnaires were included. Studies should have met the following criteria for ≥ 50% of the study sample: adult survivors (attained age ≥ 18 years) treated for any type of childhood cancer (diagnosed ≤ 21 years) and ≥ 2 years post‐diagnosis. For studies without percentages, eligibility was based on a mean age at diagnosis of 0–18 years, a mean attained age of ≥ 18 years, and a mean time since diagnosis of ≥ 2 years. In addition to all age‐related criteria, we applied a lower limit of attained age of 16 years, as children and adults may experience different worries. We excluded intervention studies without baseline CRW measurements and those that only assessed generic anxiety or did not report different CRW types separately. We used the definitions of specific worries as used by the included studies.

### 2.2 Search Strategy and Study Selection

We searched MEDLINE/PubMed and APA PsycINFO (from inception until 29–01‐2024 and 11–03‐2024, respectively). Specific worries were not pre‐defined in the search strategy, allowing us to identify not only anticipated worries but also those not initially considered. Search strategies are provided in Supplementary Tables 1 and 2. We also hand‐searched reference lists of included articles, and seven experts (psychologists and researchers) in pediatric psycho‐oncology and childhood cancer survivorship from the Princess Máxima Center were consulted to identify publications not included in the electronic databases. Suggestions were evaluated based on our inclusion criteria.

Two independent reviewers (AM, AW) identified studies meeting the inclusion criteria (first based on title/abstract and then on full text). In case of overlap between included studies in study population and outcome measures, we used the article presenting the largest sample size.

### 2.3 Data Extraction and Risk of Bias Assessment

One reviewer (AM) performed data extraction and risk of bias assessment using standardized forms, with accuracy checked by another reviewer (AW). Data was extracted on study characteristics, sample characteristics, outcome measures, and results regarding prevalence, severity, means, and associations. We used the Cochrane Childhood Cancer Risk of Bias Criteria for Observational Studies (Supplementary Table 3). The aim of this assessment was to evaluate the risk of bias in the included studies, ensuring findings were interpreted within the context of methodological rigor. It informed both the limitations section, and recommendations for future research. Discrepancies in each step of the review process were resolved through discussion or by third‐party arbitration (EvD).

### 2.4 Data Analyses

Studies were grouped by broad domains of CRW, such as health‐related, financial and interpersonal worries, and then categorized by specific worries. We summarized results descriptively, using frequencies (percentages) or mean scores with standard deviations and ranges, if available. If studies reported only severity, we calculated the prevalence by summing the percentages of all categories, except ‘no worries’. If studies reported the prevalence only for women and men separately, the total prevalence was calculated by weighing the prevalence rates by sex according to their proportions in the sample. For associated factors of CRW, depending on reported analyses, we reported Pearson correlation coefficients, odds ratios, risk ratios, or regression‐coefficients, including *p*‐values.

## 3 Results

### 3.1 Results of the Search

**FIGURE 1:**
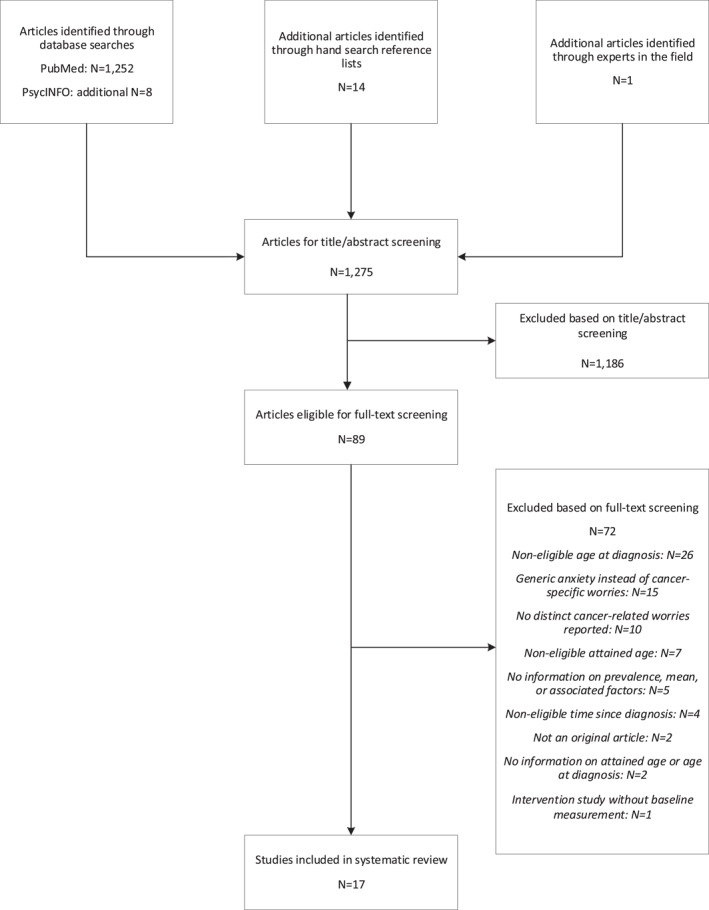
Flowchart of studies identified, screened and included in the systematic review.

A total of 1275 unique articles were identified in the initial search, of which 89 were eligible for full‐text screening. Subsequently, 72 articles were excluded because they did not meet the inclusion criteria, and 17 articles were included (Figure 1). For the worry about CCS' own children developing cancer, there was overlap between two studies [21, 22]; only the results of the study with the larger sample size were included [22]. For the worry about infertility and the worry about CCS’ own children developing cancer, there was also potential overlap in CCS between two studies [7, 22], but as the outcome measures were different, both studies were included.

### 3.2 Study Characteristics

All included data were cross‐sectional and all studies were conducted in Western countries. Sample sizes ranged from 106 [23] to 15,620 [10], with a total of 26,306 CCS. The mean/median current age, time since diagnosis, and age at diagnosis across the included studies ranged from 22.1–43.3, 8.4–31.6, and 1–10.5 years, respectively. Four studies compared worries between CCS and adults without a cancer history [22], or with siblings [10, 24, 25]. Most studies focused on CCS with mixed cancer diagnoses and treatments, but one study focused on retinoblastoma (RB) survivors [26], another on CCS who received gonadotropic treatment [27]. Two studies included only CCS with ≥ 1 children born after cancer treatment [27, 28].

### 3.3 Cancer‐Related Worries

Our review identified a range of CRW among CCS, categorized into three main themes: 1) health‐related worries, including 1a) worries about (future) health and late effects, 1b) second cancers and cancer recurrence 1c) offspring‐related worries, and 1d) death; 2) financial worries, including 2a) worries about finances; and 3) interpersonal worries, including 3a) worries about siblings, and 3b) worries about relationships. These main categories were derived using a bottom‐up approach, meaning they naturally emerged from the data based on the types of worries identified in the various studies, rather than being predetermined.

We will describe the prevalence (the percentage of CCS who experienced any level of a specific CRW), severity (the extent to which CCS experienced this worry, based on the percentage of CCS in the most severe response category), and associated factors of these worries below. In cases where studies did not provide prevalence or severity data, mean scores and scales are reported. In all instances, higher scores indicate higher levels of CRW. Most studies used single items to measure CRW, with slight differences in terminology and wording depending on the questionnaire. We adhered to the terminology and items as reported in each study, and did not merge categories unless they were identical.

For most CRW, no studies examined associated factors. Nine studies [8, 10, 24, 25, 26, 27, 29, 30, 31] investigated associated factors of specific CRW, three studies using univariable analysis only [26, 29, 31]. The significant results of only the multivariable analyses are described below.

Detailed study characteristics and other results are included in Supplementary Table 4. An overview of the main results is provided in Table 1.

**TABLE 1:** Prevalence and/or means of different cancer‐related worries in adult survivors of childhood cancer, and associated factors.

Worries	Studies	N^a^ Follow‐up time	Prevalence of worries	Mean	Factors significantly associated with worries in multivariable analysis	Risk of bias assessment^c^
Health‐related: Worries related to the survivors' health and physical well‐being						
(Future) health and late effects						
Fear/worries about (future) health	Ford et al. [26]. USA	*N*: 470 RB survivors treated in New York Follow‐u*p*: NR	Concerned: 92.1% (Somewhat/concerned/not very: 76.6%; very concerned: 15.5%)			SB: Unclear risk AB: low risk C: not applicable MB: Unclear risk
	Gibson et al. [10]. USA	*N*: 15,620 CCS Median: 17 years since diagnosis (range 14–21) Control grou*p*: 3991 siblings	Concerned: 88.1% (Not very concerned: 18.7%; Concerned: 23.2%; somewhat concerned: 21.4%; very concerned: 24.8%) Control group Significantly more worries in CCS than their siblings (95% CI: 1.09–1.15)		< 20 Gy radiation dose, location not reported (vs. none): RR: 1.1 (95% CI: 1.0–1.1); ≥ 20 Gy radiation dose, location not reported (vs. none): RR: 1.1 (95% CI: 1.1–1.2) Chest radiation (yes vs. no): RR: 1.1 (95% CI: 1.1–1.1) Neck radiation (yes vs. no): RR: 1.1 (95% CI: 1.1–1.1) Chemotherapy: Alkylating agent (vs. no alkylating agent): RR: 1.1 (95% CI: 1.0–1.1); Anthracyclines (vs. no anthracyclines): RR: 1.1 (95% CI: 1.0–1.1)	SB: Unclear risk AB: low risk C: low risk MB: High risk
	Maas et al., 2023. The Netherlands [7]	*N*: 1713 Mean: 29.2 years since diagnosis (range: 15.3–55.0)	Not at all/a little bit: 65.3% Somewhat: 21.7% Quite a bit/very much: 12.9%	Range 1–5, mean 2.2 SD 1.1		SB: High risk AB: low risk C: not applicable MB: low risk
	Cox et al., 2009. USA. [31]	*N*: 838 Mean: 21.7 years since diagnosis (≥ 5 years after treatment)		Range 1–5, mean 2.9 SD 1.1		SB: High risk AB: low risk C: not applicable MB: High risk
	Van Erp et. al. (2021). The Netherlands [32]	*N*: 151 Mean: 13.6 years since diagnosis (range 6–27)		Range 1–5, mean 2.4 SD 1.3		SB: Unclear risk AB: low risk C: not applicable MB: low risk
Worry about late effects	Kepak et al., 2022. Czech Republic [33]	*N*: 133 Mean: 16.8 years since diagnosis (range 5–43)	Concerns: 83% (some: 45%; major: 38%) Some and major concerns were not specified			SB: Unclear risk AB: low risk C: not applicable MB: Unclear risk
Worry about cancer‐related physical problems	McDonnell et al., 2021. USA. [8]	*N*: 3211 Mean: 22.8 years since diagnosis (range NR)	45% (95% CI: 43.5%–46.9%) Responses were classified as “worried” if participants responded ‘agree’ or ‘strongly agree’ on a scale ranging from 1 (strongly disagree or 5 strongly agree)		Model treatment exposures: History of chemotherapy (yes vs. no): RR: 1.2 (95% CI: 1.1–1.4) History of CRT (yes vs. no): RR: 1.4 (95% CI: 1.2–1.5) History of non‐CRT (yes vs. no): RR: 1.3 (95% CI: 1.2–1.5) Model chronic conditions: Overweight/obese (vs. underweight/normal): RR: 1.1 (95% CI: 1.0–1.2) Cardiovascular disorder (grade 3/4 vs. grade 0/1/2): RR: 1.1 (95% CI: 1.0–1.3) Sexual reproductive disorder (grade 3/4 vs. grade 0/1/2): RR: 1.3 (95% CI: 1.2–1.4) Neurologic disorder (grade 3/4 vs. grade 0/1/2): RR: 1.2 (95% CI: 1.1–1.3) Model psychological and somatic symptoms: Anxiety (yes vs. no): RR: 1.4 (95% CI: 1.2–1.5) Depression (yes vs. no): RR: 1.2 (95% CI: 1.1–1.3) Pain intensity (none to moderate to very severe bodily pain vs. a little bit of pain): RR: 1.4 (95% CI: 1.2–1.5) Pain interference (moderate to extreme pain interference vs. not at all to mild pain interference): RR: 1.2 (95% CI: 1.0–1.3)	SB: High risk AB: Unclear risk C: low risk MB: High risk
Worry about the discovery of a health problem at check‐up	Cox et al., 2009. USA. [31]	*N*: 838 Mean: 21.7 years since diagnosis (≥ 5 years after treatment)		Range 1–5, mean 2.2, SD 1.2		SB: High risk AB: low risk C: not applicable MB: High risk
Recurrence of primary malignancy and second cancers						
Worry about developing another cancer	Ford et al. [26]. USA	*N*: 470 RB survivors treated in New York Follow‐u*p*: NR	Concerned: 90.6% (somewhat/concerned/not very: 70.5%; very concerned: 20.1%)			SB: Unclear risk AB: low risk C: not applicable MB: Unclear risk
	Gibson et al. [10]. USA	*N*: 15,620 CCS Median: 17 years since diagnosis (range 14–21) Control grou*p*: 3991 siblings	Concerned: 82.8% (Not very concerned: 22.8%; Concerned: 21.1%; somewhat concerned: 18.3%; very concerned: 20.6%) Control group Proportions of worries did not differ between CCS and siblings		≥ 20 Gy radiation dose, location not reported (vs. none): RR: 1.1 (95% CI: 1.1–1.2) Chest radiation (yes vs. no): RR: 1.1 (95% CI: 1.1–1.1) Neck radiation (yes vs. no): RR: 1.1 (95% CI: 1.1–1.1) Chemotherapy: Alkylating agent (vs. no alkylating agent): RR: 1.1 (95% CI: 1.0–1.1); Anthracyclines (vs. no anthracyclines): RR: 1.1 (95% CI: 1.0–1.1)	SB: Unclear risk AB: low risk C: low risk MB: High risk
	McDonnell et al., 2021. USA. [8]	*N*: 3211 Mean: 22.8 years since diagnosis (range NR)	64% (95% CI: 62.6%–65.9%) Responses were classified as “worried” if participants responded ‘agree’ or ‘strongly agree’ on a scale ranging from 1 (strongly disagree or 5 strongly agree)		Model treatment exposures: History of non‐CRT (yes vs. no): RR: 1.1 (95% CI: 1.0–1.2) Model chronic conditions: Overweight/obese (vs. underweight/normal): RR: 1.1 (95% CI: 1.0–1.1) Cardiovascular disorder (grade 3/4 vs. grade 0/1/2): RR: 1.1 (95% CI: 1.0–1.2) Model psychological and somatic symptoms: Anxiety (yes vs. no): RR: 1.2 (95% CI: 1.1–1.3) Depression (yes vs. no): RR: 1.1 (95% CI: 1.0–1.2) Pain interference (moderate to extreme pain interference vs. not at all to mild pain interference): RR: 1.1 (95% CI: 1.1–1.2)	SB: High risk AB: Unclear risk C: low risk MB: High risk
	Langeveld et al., 2004. The Netherlands [21]	*N*: 400 attending the long‐term follow‐up clinic Mean: 16.0 years since treatment completion (≥ 5 years)	50%			SB: Unclear risk AB: low risk C: not applicable MB: low risk
	Cox et al., 2016. USA. [9]	*N*: 1189 survivors, excluding those treated at st. Jude Children's research hospital. Mean: 31.6 years since diagnosis (range 24–42)	None/not very: 39.6%; Somewhat/very: 60.4% None/not very and somewhat/very were not further specified			SB: High risk AB: low risk C: not applicable MB: High risk
Fear/worry of cancer recurrence	Kelada et al., 2019. Australia [30]	*N*: 404 Mean: 19.1 years since diagnosis (range 5–59)	Fear of cancer recurrence: 87.7% (A little or some fear of cancer recurrence: 79.0%; A lot or a great deal of fear of cancer recurrence: 8.7%)	Range 1–5, Mean: 2.3 SD 0.8	Female (vs. male): B: 0.2 (95% CI: 0.0—0.4, *p*: 0.035) Lymphoma diagnosis (vs. all other diagnoses combined): B: 0.3 (95% CI: 0.0—0.5, *p*: 0.021 More unmet needs for information about pain: B: 0.5 (95% CI: 0.2—0.8, *p*: 0.001) More unmet needs for information about fatigue: B: 0.3 (95% CI: 0.1—0.5, *p*: 0.015)	SB: Unclear risk AB: low risk C: low risk MB: Unclear risk
	Kepak et al., 2022. Czech Republic [33]	*N*: 133 Mean: 16.8 years since diagnosis (range 5–43)	Concerns: 80% (some: 40%; major: 40%) Some and major concerns were not further specified			SB: Unclear risk AB: low risk C: not applicable MB: Unclear risk
	Langeveld et al., 2004. The Netherlands [21]	*N*: 400 attending the long‐term follow‐up clinic Mean: 16.0 years since treatment completion (≥ 5 years)	54%			SB: Unclear risk AB: low risk C: not applicable MB: low risk
	McDonnell et al., 2021. USA. [8]	*N*: 3211 Mean: 22.8 years since diagnosis (range NR)	33% (95% CI: 31.2%–34.4%) Responses were classified as “worried” if participants responded ‘agree’ or ‘strongly agree’ on a scale ranging from 1 (strongly disagree or 5 strongly agree)		Model treatment exposures: History of non‐CRT (yes vs. no): RR: 1.1 (95% CI: 1.0–1.3) Relapse/second malignant neoplasm (yes vs. no): RR: 1.2 (95% CI: 1.1–1.3) Model chronic conditions: Overweight/obese (vs. underweight/normal): RR: 1.2 (95% CI: 1.1–1.3) Neurologic disorder (grade 3/4 vs. grade 0/1/2): RR: 1.2 (95% CI: 1.0–1.4) Model psychological and somatic symptoms: Anxiety (yes vs. no): RR: 1.4 (95% CI: 1.2–1.7) Depression (yes vs. no): RR: 1.2 (95% CI: 1.0–1.4) Pain intensity (none to moderate to very severe bodily pain vs. a little bit of pain): RR: 1.2 (95% CI: 1.0–1.4)	SB: High risk AB: Unclear risk C: low risk MB: High risk
	Fisher et al., 2020. USA. [29]	*N*: 114 CCS visiting the survivorship clinic Mean: 23.8 years since diagnosis (range NR)	Fear of recurrence in the past week Any number of days: 25% (several days: 18%; more than half the days: 7%; nearly every day: 0%)			SB: Unclear risk AB: low risk C: not applicable MB: High risk
	Ford et al. [26]. USA	*N*: 470 RB survivors treated in New York Follow‐u*p*: NR		Range 22–110 Survivors of unilateral RB: Mean 42.8 SD 10.6. Survivors of bilateral RB: Mean 50.2 SD 10.8.		SB: Unclear risk AB: low risk C: not applicable MB: Unclear risk
	Cox et al., 2009. USA. [31]	*N*: 838 Mean: 21.7 years since diagnosis (≥ 5 years after treatment)		Range 1–5, mean 2.4 SD 1.2		SB: High risk AB: low risk C: not applicable MB: High risk
	Berg et al., 2016. USA. [23]	*N*: 106 Mean: 8.4 years since diagnosis (range NR)		Range 0–4, mean 1.7 SD 1.4		SB: Unclear risk AB: Unclear risk C: not applicable MB: High risk
Offspring‐related						
Worries about infertility/the ability to have children	Kepak et al., 2022. Czech Republic [33]	*N*: 133 Mean: 16.8 years since diagnosis (range 5–43)	68% (some: 30%; major 38%) Some and major concerns were not specified			SB: Unclear risk AB: low risk C: not applicable MB: Unclear risk
	Cherven et al., 2022. USA. [27]	*N*: 249 cancer survivors with a history of gonadotropic treatment Mean: 8.0 years since treatment completion (range 1.0–21.5)	66.3%		Female versus male sex: OR: 2.6 (95% CI: 1.4–5.0, *p*: 0.002) Solid tumor diagnosis versus leukemia: OR: 2.3 (95% CI: 1.2–4.7, *p*: 0.019) Moderate risk of infertility versus low risk: OR: 2.9 (95% CI: 1.2–7.6, *p*: 0.02) High risk of infertility versus low risk: OR: 3.3 (95% CI: 1.6–7.2, *p*: 0.002). Treatment‐related risk for infertility (low, moderate, or high risk) was determined based on each survivor's gonadotropic exposures. ≥ 2 versus 1 fertility discussions during survivorship care: OR: 2.7 (95% CI: 1.5–5.2, *p*: 0.002)	SB: Unclear risk AB: low risk C: high risk MB: High risk
	Langeveld et al., 2003. The Netherlands [22]	*N*: 500 Mean: 15 years since treatment completion (range 5–33) Control grou*p*: 1092 young adults without a cancer history	58.6% Control group Significantly more worries in CCS than in adults without cancer history (p < 0.001)			SB: Unclear risk AB: low risk C: not applicable MB: High risk
	Ford et al. [26]. USA	*N*: 470 RB survivors treated in New York Follow‐u*p*: NR	33.7% (somewhat/concerned/not very: 24.5%; very concerned: 9.2%)			SB: Unclear risk AB: low risk C: not applicable MB: Unclear risk
	Cox et al., 2016. USA. [9]	*N*: 1189 survivors, excluding those treated at st. Jude Children's research hospital. Mean: 31.6 years since diagnosis (range 24–42)	Not at all/not very much: 73.4% Somewhat/very much: 26.6% Not at all/not very much, and somewhat/very much were not further specified			SB: High risk AB: low risk C: not applicable MB: High risk
	Maas et al., 2023. The Netherlands [7]	*N*: 1713 Mean: 29.2 years since diagnosis (range: 15.3–55.0)	Not at all/a little bit: 71.4% Somewhat: 12.7% Quite a bit/very much: 15.9%	Range 1–5, mean 1.9 SD 1.3		SB: High risk AB: low risk C: not applicable MB: low risk
Worry about children getting cancer	Langeveld et al., 2003. The Netherlands [22] ^b^	*N*: 500 Mean: 15 years since treatment completion (range 5–33) Control grou*p*: 1092 young adults without a cancer history	45.2% Control group Significantly more worries in CCS than in adults without cancer history (p < 0.001)			SB: Unclear risk AB: low risk C: not applicable MB: High risk
	Ford et al. [26]. USA	*N*: 470 RB survivors treated in New York Follow‐u*p*: NR	Worry about possibility of children getting RB Never/rarely: 47.7% Sometimes/often: 52.3% Never/rarely and sometimes/often were not further specified			SB: Unclear risk AB: low risk C: not applicable MB: Unclear risk
	Dalkner et al., 2023. Austria, Czech Republic, Germany, Poland, Switzerland [24]	*N*: 256 CCS who have ≥ 1 children born after cancer treatment Mean: 28.1 years since diagnosis (range NR) Control grou*p*: 256 siblings	Fear of possible cancer development in offspring on a 10 cm long visual scale None/low (<2 cm): 36.7%; Medium (2–6 cm): 36.7%; High/very high (>6 cm): 26.6% Control group CCS higher levels of fear of possible cancer development in offspring than their siblings (*p*: 0.044)	Range 0–10, mean: 4.10 SD 2.96	Partial correlation Younger age of parent (current): r: −0.2, *p*: 0.014 Shorter time since therapy: r: −0.2, *p*: 0.003 Lower number of children: r: −0.2, *p*: 0.001	SB: Unclear risk AB: low risk C: low risk MB: High risk
	Balcerek et al., 2015. Germany [28]	*N*: 254 CCS who have ≥ 1 children born after cancer treatment Mean years since diagnosis: NR (≥ 6 years)	Fear of possible cancer development in offspring on a 10 cm long visual scale No to very low (0–20.0 mm): 26.5%; Low (20.1–40.0 mm): 17.3%; Moderate (40.1–60.0 mm): 20.8%; High (60.1–80.0 mm): 16.1%; Very high (80.1–100.0 mm): 19.3%			SB: Unclear risk AB: low risk C: not applicable MB: High risk
	Maas et al., 2023. The Netherlands [7]	*N*: 1713 Mean: 29.2 years since diagnosis (range: 15.3–55.0)	Not at all/a little bit: 69.3% Some impact: 17.2% Quite a bit/very much: 13.5%	Range 1–5, Mean 2.0 SD 1.2		SB: High risk AB: low risk C: not applicable MB: low risk
Worry about health of children	Balcerek et al., 2015. Germany [28]	*N*: 254 CCS who have ≥ 1 children born after cancer treatment Mean years since diagnosis: NR (≥ 6 years)	19.9%			SB: Unclear risk AB: low risk C: not applicable MB: High risk
	Maas et al., 2023. The Netherlands [7]	*N*: 1713 Mean: 29.2 years since diagnosis (range: 15.3–55.0)	Not at all/a little bit: 68.2% Somewhat: 16.9% Quite a bit/very much: 14.9%	Range 1–5, mean 2.1 SD 1.2		SB: High risk AB: low risk C: not applicable MB: low risk
Death						
Afraid to die	Maas et al., 2023. The Netherlands [7]	*N*: 1713 Mean: 29.2 years since diagnosis (range: 15.3–55.0)	Not at all/a little bit: 78.6% Somewhat: 11.2% Quite a bit/very much: 10.1%	Range 1–5, mean 1.8 SD 1.1		SB: High risk AB: low risk C: not applicable MB: low risk
	Van Erp et. al. (2021). The Netherlands [32]	*N*: 151 Mean: 13.6 years since diagnosis (range 6–27)		Range 1–5, mean 2.1 SD 1.4		SB: Unclear risk AB: low risk C: not applicable MB: low risk
Worry about dying at a young age	Maas et al., 2023. The Netherlands [7]	*N*: 1713 Mean: 29.2 years since diagnosis (range: 15.3–55.0)	Not at all/a little bit: 81.0% Somewhat: 11.1% Quite a bit/very much: 7.9%	Range 1–5, mean 1.7 SD 1.1		SB: High risk AB: low risk C: not applicable MB: low risk
	Van Erp et. al. (2021). The Netherlands [32]	*N*: 151 Mean: 13.6 years since diagnosis (range 6–27)		Range 1–5, mean 2.0 SD 1.3		SB: Unclear risk AB: low risk C: not applicable MB: low risk
Financial: Worries about the financial implications of cancer						
Finances and insurance						
Worries about insurance coverage	Fair et al., 2021. USA. [25]	*N*: 698 of which 10.2% (n: 79) uninsured Median: 28.8 years since diagnosis (range 23.1–41.7) Control grou*p*: 210 siblings of which 7.9% (n: 21) uninsured	Instances of worries: 54.5% (1–2 instances: 22.8%; ≥ 3 instances: 31.7%) Control group Among insured, worries about insurance coverage not different between CCS and siblings		(≥ 3 vs 0‐2 instances), insured only: Female versus male sex: OR: 1.9 (95% CI: 1.3–2.9, *p*: 0.002) Younger age (0–4 years) at diagnosis (vs. 5–20 years): OR: 0.6 (95% CI: 0.4–0.9, *p*: 0.02) ≥ 30 years since diagnosis (vs. 21–29 years): OR: 1.7 (95% CI: 1.1–2.5, *p*: 0.01)	SB: High risk AB: low risk C: low risk MB: Unclear risk
Worries about finances	Fair et al., 2021. USA. [25]	*N*: 698 of which 10.2% (n: 79) uninsured Median: 28.8 years since diagnosis (range 23.1–41.7) Control grou*p*: 210 siblings of which 7.9% (n: 21) uninsured	Instances of worries: 43.2% (1–2 instances: 26.5%; ≥ 3 instances: 16.7%) Control group Proportions not significantly different between CCS and siblings		(≥ 3 vs 0‐2 instances): Female versus male sex: OR: 2.2 (95% CI: 1.3–3.7, *p*: 0.003) Non‐hispanic white ethnicity versus other: OR: 0.4 (95% CI: 0.2–0.9, *p*: 0.03) Diagnosis leukemia versus bone tumor: OR: 3.1 (95% CI: 1.1–9.3, *p*: 0.04) Younger age (0–4 years) at diagnosis versus 5–20 years: OR: 0.4 (95% CI: 0.2–0.8, *p*: 0.004) Insured status (vs. non‐insured): OR: 0.1 (95% CI: 0.1–0.2, p < 0.001)	SB: High risk AB: low risk C: low risk MB: Unclear risk
Worry about the ability to get health insurance	Cox et al., 2016. USA. [9]	*N*: 1189 survivors, excluding those treated at st. Jude Children's research hospital. Mean: 31.6 years since diagnosis (range 24–42)	Not at all/not very much: 64.2% Somewhat/very much: 35.8% Not at all/not very much and somewhat/very much were not further specified			SB: High risk AB: low risk C: not applicable MB: High risk
Interpersonal: Worries about the impact of the cancer on siblings and relationships						
Siblings						
Worries about impact cancer on siblings	Maas et al., 2023. The Netherlands [7]	*N*: 1713 Mean: 29.2 years since diagnosis (range: 15.3–55.0)	Not at all/a little bit: 65.5% Somewhat: 19.1% Quite a bit/very much: 15.4%	Range 1–5, mean 2.1 SD 1.2		SB: High risk AB: low risk C: not applicable MB: low risk
	Van Erp et. al. (2021). The Netherlands [32]	*N*: 151 Mean: 13.6 years since diagnosis (range 6–27)		Range 1–5, mean 2.8, SD 1.4		SB: Unclear risk AB: low risk C: not applicable MB: low risk
Relationships						
Partnered						
Worry about having sex with partner	Maas et al., 2023. The Netherlands [7]	*N*: 1713 Mean: 29.2 years since diagnosis (range: 15.3–55.0)	Not at all/a little bit: 88.1% Somewhat: 5.9% Quite a bit/very much: 6.0%	Range 1–5, mean 1.4 SD 0.9		SB: High risk AB: low risk C: not applicable MB: low risk
	Van Erp et. al. (2021). The Netherlands [32]	*N*: 151 Mean: 13.6 years since diagnosis (range 6–27)		Range 1–5, mean 1.4, SD 0.7		SB: Unclear risk AB: low risk C: not applicable MB: low risk
Worry partner will leave if cancer returns	Maas et al., 2023. The Netherlands [7]	*N*: 1713 Mean: 29.2 years since diagnosis (range: 15.3–55.0)	Not at all/a little bit: 91.2% Somewhat: 5.2% Quite a bit/very much: 3.6%	Range 1–5, mean 1.3 SD 0.8		SB: High risk AB: low risk C: not applicable MB: low risk
	Van Erp et. al. (2021). The Netherlands [32]	*N*: 151 Mean: 13.6 years since diagnosis (range 6–27)		Range 1–5, mean 1.7, SD 1.2		SB: Unclear risk AB: low risk C: not applicable MB: low risk
Unpartnered						
Worry about having no relationship	Maas et al., 2023. The Netherlands [7]	*N*: 1713 Mean: 29.2 years since diagnosis (range: 15.3–55.0)	Not at all/a little bit: 59.2% Somewhat: 21.4% Quite a bit/very much: 19.4%	Range 1–5, mean 2.3 SD 1.3		SB: High risk AB: low risk C: not applicable MB: low risk
	Van Erp et. al. (2021). The Netherlands [32]	*N*: 151 Mean: 13.6 years since diagnosis (range 6–27)		Range 1–5, mean 2.2 SD 1.2		SB: Unclear risk AB: low risk C: not applicable MB: low risk
Worry about telling potential partner about fertility	Maas et al., 2023. The Netherlands [7]	*N*: 1713 Mean: 29.2 years since diagnosis (range: 15.3–55.0)	Not at all/a little bit: 71.7% Somewhat: 12.1% Quite a bit/very much: 16.2%	Range 1–5, mean 2.0 SD 1.3		SB: High risk AB: low risk C: not applicable MB: low risk
	Van Erp et. al. (2021). The Netherlands [32]	*N*: 151 Mean: 13.6 years since diagnosis (range 6–27)		Range 1–5, mean 2.2 SD 1.3		SB: Unclear risk AB: low risk C: not applicable MB: low risk
Worry about having sex	Maas et al., 2023. The Netherlands [7]	*N*: 1713 Mean: 29.2 years since diagnosis (range: 15.3–55.0)	Not at all/a little bit: 80.1% Somewhat: 10.8% Quite a bit/very much: 9.1%	Range 1–5, mean 1.7 SD 1.1		SB: High risk AB: low risk C: not applicable MB: low risk
	Van Erp et. al. (2021). The Netherlands [32]	*N*: 151 Mean: 13.6 years since diagnosis (range 6–27)		Range 1–5, mean 1.8 SD 1.2		SB: Unclear risk AB: low risk C: not applicable MB: low risk
Worry about telling potential partner about childhood cancer	Maas et al., 2023. The Netherlands [7]	*N*: 1713 Mean: 29.2 years since diagnosis (range: 15.3–55.0)	Not at all/a little bit: 83.0% Somewhat: 8.4% Quite a bit/very much: 8.6%	Range 1–5, mean 1.6 SD 1.0.		SB: High risk AB: low risk C: not applicable MB: low risk
	Van Erp et. al. (2021). The Netherlands [32]	*N*: 151 Mean: 13.6 years since diagnosis (range 6–27)		Range 1–5, mean 1.6 SD 0.9		SB: Unclear risk AB: low risk C: not applicable MB: low risk

*Note:* Due to rounding, some significant results may appear non‐significant because values close to 1 are reported as exactly 1. All reported results are significant.

^a^ Various cancer diagnoses and treatment types, unless otherwise specified.

^b^ Worry about children getting cancer was reported by both Langeveld (2003) and Langeveld (2004). However, due to the overlap in study populations, only the results from Langeveld (2003) are presented, as this study included the larger sample size.

^c^ Risk of bias assessment: SB=Selection Bias, AB=Attrition Bias, C=Confounding, MB=Measurement Bias. Detailed information, including the rationale for each assessment, is provided in Supplementary Table 4. Abbreviations NR=Not reported, CCS = childhood cancer survivors, SD=Standard Deviation, RR=Risk Ratio, OR=Odds Ratio, SCT=Stem Cell Transplantation, BMT=Bone Marrow Transplantation, SMN=Second Malignant Neoplasm, CRT=Cranial Radiation Therapy, RB=retinoblastoma, Gy=Gray.

#### 3.3.1 1. Health‐Related

##### 3.3.1.1 Worries About (Future) Health and Late Effects

###### 3.3.1.1.1 Fear or Worries About (Future) Health

Prevalence: In two studies [10, 26], including 16,090 CCS, the prevalence of CCS’ fear/worry about their (future) health ranged from 88.1% to 92.1%. The study with the 92.1% prevalence was specific to RB survivors.

Severity: Three studies [7, 10, 26], including 17,803 CCS, examined severity. One study [10] observed that, while 88.1% of CCS were worried, 24.8% were very worried. In another study [7], 12.9% of CCS were quite a bit to very much worried. The third study [26] focused on RB survivors, and found that 92.1% were worried, and 15.5% were very worried. One study [10] found, comparing proportions of worries, that CCS worried more about their health than their siblings (95% CI: 1.09–1.15).

Mean: Two studies [31, 32], including 989 CCS, only reported mean scores. They observed that, on average, CCS scored between 2.4–2.9 on a 1‐5 scale.

Associated Factors: One study [10], including 15,620 CCS, addressed associated factors, and showed that specific chemotherapy types, namely alkylating agent (vs. no alkylating agent), and anthracyclines (vs. no anthracyclines) were associated with more worries. Also, both < 20 Gy and ≥ 20 Gy radiation dose (vs. none), chest and neck radiation (yes vs. no) were associated with more worries.

###### 3.3.1.1.2 Worry About Late Effects

Prevalence: In one study [33], including 133 CCS, the prevalence of worry about late effects was 83%.

Severity: While 83% of CCS worried about late effects, 38% of CCS had major worries [33].

###### 3.3.1.1.3 Worry About Cancer‐Related Physical Problems

Prevalence: In one study [8], including 3211 CCS, the prevalence of worry about cancer‐related physical problems was 45%.

Associated Factors: This study also observed that chemotherapy, cranial radiotherapy, and non‐cranial radiotherapy (all: yes vs. no), being overweight/obese (vs. underweight/normal), having a cardiovascular, sexual reproductive or neurologic disorder (all: grade 3/4 vs. grade 0/1/2), anxiety and depression (yes vs. no), pain intensity (none to moderate to very severe bodily pain vs. a little bit of pain), and pain interference (moderate to extreme pain interference vs. not at all to mild pain interference) were associated with more worries.

###### 3.3.1.1.4 Worry About the Discovery of a Health Problem at Check‐Up

No studies investigated the prevalence of worry about the discovery of a health problem at check‐up.

Mean: One study [31] including 838 CCS, observed that, on average, CCS scored 2.23 on a 1‐5 scale.

##### 3.3.1.2 Worry About Second Cancers and Cancer Recurrence

###### 3.3.1.2.1 Worry About Developing Another Cancer

Prevalence: In four studies [8, 10, 21, 26], including 19,701 CCS, the prevalence of CCS’ worry about developing another cancer ranged from 50% to 90.6%. The study observing a 90.6% prevalence was in RB survivors only [26].

Severity: Three studies [9, 10, 26], including 17,279 CCS, examined severity. One study [9] observed that 60.4% were somewhat to very worried. Another study [10] found that, while 82.8% of CCS reported worries, 20.6% were very worried. The third study [26] noted that, while 90.6% of RB survivors reported worries, 20.1% were very worried. One study [10] found that proportions of worries about developing cancer did not differ between CCS and siblings.

Associated Factors: Two studies [8, 10], including 18,831 CCS, explored factors associated with worry about developing another cancer. One [8] found that non‐cranial radiotherapy (yes vs. no), being overweight/obese (vs. underweight/normal), having a cardiovascular disorder (grade 3/4 vs. grade 0/1/2), anxiety and depression (yes vs. no), and pain interference (moderate to extreme pain interference vs. not at all to mild pain interference) were associated with more worries. While this study did not find a significant association between chemotherapy and more worries, another study [10] showed that specific chemotherapy types, namely alkylating agent (vs. no alkylating agent), and anthracyclines (vs. no anthracyclines) were associated with more worries. Also, ≥ 20 Gy radiation dose (vs. none), chest and neck radiation (yes vs. no) were associated with more worries. In both studies, no associations with cranial radiation therapy were found.

###### 3.3.1.2.2 Fear or Worry About Cancer Recurrence

Prevalence: In five studies [8, 21, 29, 30, 33], including 4262 CCS, the prevalence of CCS’ fear/worry about cancer recurrence varied from 25% to 87.7%.

Severity: Three studies [29, 30, 33], including 651 CCS, examined severity. One [30] found that, while 87.7% of CCS reported fear of cancer recurrence, 8.7% had a lot or great deal of fear of cancer recurrence. Another [33] found that 80% of CCS reported worries, and 40% had major worries. The third study [29] observed that 25% of CCS experienced fear of cancer recurrence in the past week, but none experienced this nearly every day.

Mean: Three studies [23, 26, 31], including 1414 CCS, examined only mean scores of fear of cancer recurrence. One [31] reported a mean of 2.4 on a 1‐5 scale, another [23] reported a mean of 1.7 on a 0‐4 scale, and the third one [26] reported a mean of 42.8–50.2 on a 22‐110 scale in RB survivors.

Associated Factors: Two studies [8, 30], including 3615 CCS, explored factors associated with fear/worry of cancer recurrence. One [30] observed that female sex, lymphoma diagnosis (vs. any other diagnosis), and more unmet needs for information about pain and fatigue were associated with more fear of cancer recurrence. However, no associations were found between more pain and fatigue themselves and worries. Another study [8] adjusted for sex in the analysis (results for sex not reported), and found that non‐cranial radiotherapy (yes vs. no), relapse or second malignancy (yes vs. no), being overweight/obese (vs. underweight/normal), neurological disorders (grade 3/4 vs. grade 0/1/2), anxiety (yes vs. no), depression (yes vs. no), and pain (none to moderate to very severe bodily pain vs. a little bit of pain) were associated with more worries.

##### 3.3.1.3 Offspring‐Related Worries

###### 3.3.1.3.1 Worries About Infertility

Prevalence: In four studies [22, 26, 27, 33], including 1352 CCS, the prevalence of infertility worries varied between 33.7% and 68%. Two of these studies focused on subgroups. One [27] reported a prevalence of 66.3% among those with a history of gonadotropic treatment, another [26] observed 33.7% among RB survivors. One study [22] showed that CCS worried more about infertility than adults without a cancer history (*p* < 0.001).

Severity: Four studies [7, 9, 26, 33], including 3505 CCS, examined severity. One study [33] found that, while 68% of CCS reported infertility worries, 38% had major worries. Another study [9] reported that 26.6% were somewhat to very much worried, and a third study [7] observed that 15.9% were quite a bit to very much worried. The fourth study [26] found that, while 33.7% of RB survivors reported worries, 9.2% were very worried.

Associated Factors: One study [27], including 249 CCS, investigated factors associated with worry about infertility. This study found that female sex, a solid tumor diagnosis (vs. leukemia), moderate to high infertility risk (vs. low risk), and having ≥ 2 fertility discussions during survivorship care (vs. 1 discussion) were associated with more worries.

###### 3.3.1.3.2 Worries About Children Getting Cancer

Prevalence: In one study [22], including 500 CCS, the prevalence of CCS’ worries about their own children getting cancer, was 45.2%. Additionally, it demonstrated that CCS were more worried than adults without a history of cancer about their children getting cancer (*p* < 0.05).

Severity: Four studies [7, 24, 26, 28], including 2693 CCS, examined severity. One study [7] observed that 13.5% of CCS were quite a bit to very much worried. Three studies focused on subgroups of CCS, one [26] observed that 52.3% of RB survivors sometimes or often worried about their children getting RB. Two studies [24, 28] in CCS with ≥ 1 child(ren) born after cancer treatment reported that 26.6%–35.4% had a high or very high degree of worry. One study [24] showed that CCS had higher levels of fear of cancer development in their children than their siblings (*p* < 0.001).

Associated Factors: One study [24], including 256 CCS with ≥ 1 child(ren) born after cancer treatment, examined factors associated with CCS’ worries about children getting cancer. Younger age of the CCS, shorter time since therapy, and a lower number of children were associated with more worries.

###### 3.3.1.3.3 Worries About the Health of Children

Prevalence: One study [28], including 254 CCS with ≥ 1 child(ren) born after cancer treatment, investigated the prevalence of CCS’ worries regarding the health of their own children, and observed that 19.9% of CCS worried about this.

Severity: One study [7], including 1713 CCS, examined severity, and indicated that 14.9% of CCS were quite a bit to very much worried.

##### 3.3.1.4 1d) Worries About Death

###### 3.3.1.4.1 Being Afraid to die and Worry About Dying at a Young Age

No studies investigated the prevalence of worries about dying. However, one study [7], including 1713 CCS, reported the severity of these worries, and two studies [7, 32], including 1864 CCS, reported mean scores.

Severity: Respectively 10.1% and 7.9% of CCS were quite a bit to very much afraid of dying and of dying at a young age [7].

Mean: On average, CCS scored between 1.8–2.1 and between 1.7–2.0 on a 1‐5 scale regarding these specific worries [7, 32].

#### 3.3.2 Financial

##### 3.3.2.1 Worries About Finances

###### 3.3.2.1.1 Worries About Insurance Coverage and Finances

One study [25], including 698 CCS, of whom 10.2% were uninsured, examined worries about both insurance coverage and finances.

Prevalence: Among these CCS, 54.5% had experienced ≥ 1 instances of worry about insurance coverage, and 43.2% experienced ≥ 1 instances of financial worries.

Severity: While 54.5% of CCS reported ≥ 1 instances of insurance worries, 31.7% of CCS experienced ≥ 3 instances. Additionally, while 43.2% of CCS reported ≥ 1 instances of financial worries, 16.7% of CCS experienced ≥ 3 instances. For both worries, proportions did not differ between CCS and siblings.

Associated Factors: Female sex, younger age at diagnosis (0–4 vs. 5–20 years), and being ≥ 30 years post‐diagnosis (vs. 21–29 years) were associated with more instances of worries about insurance coverage. Additionally, female sex, non‐Hispanic white ethnicity (vs. other), leukemia diagnosis (vs. bone tumor), younger age at diagnosis (0–4 vs. 5–20 years), and insured status (vs. non‐insured) were associated with more instances of financial worries.

##### 3.3.2.2 Worry About Obtaining Health Insurance

No studies investigated the prevalence of the worry about obtaining health insurance.

Severity: One study [9], including 1189 CCS, examined the severity of this worry, and observed that 35.8% were somewhat to very much worried.

#### 3.3.3 Interpersonal

##### 3.3.3.1 Worries About Siblings

###### 3.3.3.1.1 Worry About the Impact of Childhood Cancer on Siblings

No studies investigated the prevalence of worries about the impact of childhood cancer on siblings. However, one study [7], including 1713 CCS, reported the severity of these worries and two studies [7, 32], including 1864 CCS, reported mean scores.

Severity: Among CCS, 15.4% were quite a bit to very much worried about the impact of childhood cancer on their siblings.

Mean: On average, CCS scored between 2.1–2.8 on a 1‐5 scale regarding this worry.

#### 3.3.4 Worries About Relationships

No studies investigated the prevalence of relationship worries. However, one study [7], including 1713 CCS, reported the severity of these worries, and two studies [7, 32], including 1864 CCS, reported mean scores.

##### 3.3.4.1 Worries Among CCS With a Partner

Severity: Among CCS with a partner, 3.6%–6.0% were quite a bit to very much worried about having sex with their partner and about their partner leaving if the cancer would return.

Mean: On average, CCS scored between 1.4–1.7 on a 1‐5 scale for worries about having sex with their partner and for worries about their partner leaving if the cancer would return.

#### 3.3.5 Worries Among CCS Without a Partner

Severity: Among CCS without a partner, 19.4% were quite a bit to very much worried about not having a relationship, and 16.2% about telling a potential partner about infertility. Lower percentages (8.6%–9.1%) were observed for quite a bit to very much worry about having sex and telling a potential partner about childhood cancer.

Mean: On average, CCS scored between 1.3–2.2 on a 1‐5 scale for the worry about telling a potential partner about infertility, having no relationship, having sex, and telling a potential partner about childhood cancer.

Risk of Bias Assessment: For detailed information, see Supplementary Table 4.

In 76.5% of studies, the risk of selection bias was unclear due to unknown original cohorts [10, 21, 22, 23, 24, 25, 26, 27, 28, 29, 30, 32, 33]. In 23.5%, risk was high due to low participation rates [7, 8, 9, 31].

In 88.2% of studies the risk of attrition bias was low, with over 75% of CCS having CRW assessments [7, 9, 10, 21, 22, 24, 25, 26, 27, 28, 29, 30, 31, 32, 33]. Risk was unclear in 11.8% of studies [8, 23].

Of 9 studies assessing associated factors, 55.6% adjusted for all key factors and were low risk [8, 10, 24, 25, 30]; 44.4% were high risk: either for not adjusting for attained age in the multivariable analysis [27] or due to univariable analyses [26, 29, 31].

In 58.8% of studies the risk of measurement bias was high due to the use of unvalidated single items [8, 9, 10, 22, 23, 24, 27, 28, 29, 31]. Risk was unclear in 23.5% of the studies, which used adapted versions of standardized measures that were possibly unvalidated [25, 26, 30, 33]. The risk was low in 17.6% with validated standardized questionnaires [7, 21, 32].

## 4 Discussion

This systematic review provides the first comprehensive overview of CRW in adult CCS. The review included 17 studies with a total of 26,306 CCS.

### 4.1 Main Findings

Our review identified a range of CRW among CCS, categorized into three main themes: 1) health‐related worries, including 1a) worries about (future) health and late effects, 1b) second cancers and cancer recurrence 1c) offspring‐related worries, and 1d) death; 2) financial worries, including 2a) worries about insurance coverage and finances; and 3) interpersonal worries, including 3a) those about the impact of cancer on siblings, and 3b) worries about relationships.

Most prevalent were health‐related worries about (future) health (88%–92%) [10, 26], second cancers (50%–91%) [8, 10, 21, 26], cancer recurrence (25%–88%) [8, 21, 29, 30, 33], late effects (83%) [33], and infertility (34%–68%) [22, 26, 27, 33]. Variability in prevalence may result from various factors. First, the timing of assessment: some studies assessed CRW in CCS visiting survivorship clinics [21, 29], a time at which CRW are known to peak [34, 35]. Second, some studies focused on specific subgroups, like RB survivors [26], who may worry more about their children developing the same disease due to genetic predisposition, or CCS who received gonadotropic treatment [27], who likely have increased infertility worries as their treatment may impair reproductive ability [36]. Third, most studies used single items to measure CRW, which may lead to varied interpretations due to differences in wording. For instance, some studies assessed fear of cancer recurrence without specifying whether it included second cancers, while others distinguished between recurrence of the primary cancer and the development of second cancers.

On average, CCS scored on the lower half of rating scales (typically ranging from 1 = not at all worried to 5 = very much worried) for most CRW, yet a significant proportion of CCS experienced high levels of these worries [7, 24, 30, 31, 32]. Reporting the proportion of CCS with severe CRW is crucial for clinical practice, as it reveals the burden experienced by a substantial percentage of CCS, who are more likely to need targeted psychosocial care.

Although worries about infertility and the health of one's own children are present in the general population, CCS worry significantly more about these issues than adults without a cancer history [22]. CCS also have higher levels of worry about their own children developing cancer than their siblings [24]. However, there were no differences in financial or insurance worries between CCS and siblings [25]. Interestingly, while CCS worried more about their health, they did not worry more about developing cancer than siblings [10], possibly due to siblings' close experience with the illness of their brother or sister, or shared genetic predispositions.

Several factors were associated with more CRW. Female sex was associated with worries about cancer recurrence, infertility, insurance coverage, and finances [25, 27, 30]. In adult‐onset cancer survivors, female sex has also consistently shown associations with more CRW [18, 19, 37]. Factors such as pain‐related variables [8, 30], mood‐related variables [8], chronic health conditions [8], and treatment history [8, 10] were also associated with several CRW, including cancer‐related physical problems and the fear of cancer recurrence and second cancers. Pain [38], chronic health conditions [39], and treatment histories [40], represent or can lead to health problems, which in turn may trigger worries.

Time since diagnosis was not typically associated with CRW [25, 27, 30], although one study [24] observed a slight negative association with the fear of cancer development in offspring. It is possible that CRW remain stable over time, as reported in the literature for several CRW [10, 16, 17].

There are limited theoretical models available to understand CRW and their associated factors. Lee‐Jones et al. [41] proposed the Self‐Regulatory Executive Function (S‐REF) model, which suggests that fear of cancer recurrence is triggered by both internal (e.g., somatic cues, interpreted as symptoms) and external (e.g., hospital appointments, media exposure, and family concerns) stimuli. These stimuli activate cognitive responses and emotional reactions, including worrying thoughts about recurrence and anxiety about cancer itself, leading to psychological effects such as misinterpretation of symptoms and increased somatic anxiety. Despite growing interest in CRW, the theoretical framework first proposed by Lee‐Jones et al. has received limited attention and has yet to be tested. The development and evaluation of theoretical models for CRW should therefore be prioritized to advance our understanding of this important outcome in CCS.

### 4.2 Study Limitations

Some issues should be considered when interpretating the results. Firstly, all studies were cross‐sectional, which limited our ability to track the development of CRW over time, and to establish cause and effect in identified associations. Secondly, our review focused on quantitative studies, thereby excluding open‐ended exploration of CRW. However, qualitative studies on this topic have identified similar CRW [15, 23, 42, 43, 44, 45]. Thirdly, this study focuses on long‐term adult CCS. Caution is advised when generalizing the results to younger CCS, as their understanding of illness and concepts like cancer recurrence and its likelihood, may differ [46]. Additionally, children may have different worries, such as worries about returning to school [47, 48]. Fourthly, all studies in this review were conducted in developed regions (North America, Europa, and Australia). Caution is advised when generalizing the results to other areas. Fifthly, the risk of selection bias in the studies was either unclear or high, which may limit the representativeness of our findings across the total population of eligible CCS. Sixthly, factors associated with CRW were identified from studies that adjusted for important prognostic factors including sex and attained age, enhancing the robustness of our findings. However, we cannot rule out that relevant factors were not identified because of low power of the studies. The same applies to differences between CCS and controls. This could potentially be resolved by conducting a meta‐analysis based on individual participant data from the original studies. Finally, most studies used single items to measure CRW, potentially not capturing all important aspects of severe CRW, like the persistence of symptoms and the impact on daily life [49]. The few studies that used validated measures lacked clinical cut‐offs for severe CRW, which have not been established for most worries.

### 4.3 Clinical Implications

CRW are prevalent among CCS and can negatively impact their mental and physical health [11, 12, 13, 50]. Therefore, it is crucial to address these worries. Measures to identify CCS with severe CRW can help determine who may benefit from help with dealing with CRW [51]. Some authors suggest to screen cancer survivors for CRW [52], while others recommend brief psycho‐education, given the high prevalence [19]. Psycho‐education could normalize low levels of CRW, provide guidance on seeking support, and teach strategies for managing cognitive processes like rumination, and attentional bias [53].

Research [9] indicates that CCS with moderate to extreme CRW often have unmet needs, particularly in managing worry and uncertainty about the future. This underscores the need for targeted psychosocial support. Most CRW interventions have focused on fear of cancer recurrence in adult‐onset cancer survivors. Various psychological and physical interventions like cognitive behavioral therapy (CBT), relaxation techniques, meditation, physical exercise [53, 54] and Acceptance and Commitment Therapy‐based (ACT) interventions [55], have shown promise in mitigating fear of cancer recurrence in this population. These methods could also help CCS by reducing distress and maladaptive coping behaviors that lead to over‐ or under‐utilization of healthcare [14]. Blended interventions in primary care could address the need for easily accessible and inexpensive support for moderate CRW, complementing existing specialized care [56].

Healthcare providers involved in survivorship care play a crucial role in discussing CRW with CCS. They should be attentive to both the presence and severity of CRW and the specific support needs of CCS. Prior research indicates that CCS have several unmet information needs, particularly about late effects and cancer recurrence [57]. CCS may not recall information discussed during childhood or interpret it differently as adults. For instance, some CCS (31%) worry about infertility or second tumors without being at actual risk [52], possibly due to inadequate knowledge. Regular repetition and reassessment of their knowledge can help mitigate unnecessary worries. Additionally, providing information about pain management is crucial, as pain and unmet needs in this area are associated with several CRW [8, 30].

While providing information about late effects is crucial, it may also heighten worries. When communicating about late effects, it can be helpful to emphasize the importance of proactive health management strategies. These include attending follow‐up clinics for regular screenings, leading a healthy lifestyle, and staying vigilant about physical symptoms. This approach can empower CCS to actively manage their health, ensuring they are informed without becoming overly alarmed.

### 4.4 Future Research

To address the gaps identified in this systematic review, future studies should focus on several key areas. First, despite the broad range of worries identified, there are likely additional worries among CCS that were not identified. For example, fears related to medical procedures and needle phobia were not identified in this study, but they may still be relevant among CCS due to the painful medical procedures many underwent during their cancer treatment. Once established, severe needle fear can follow a stable, chronic course and may persist for decades if left untreated [58], and as such, may continue to affect survivors in the long term. Additionally, with advances in genetic testing, it becomes increasingly relevant to examine worries about cancer development in family members of CCS [59]. Furthermore, worries could relate to life domains such as school, work, and physical appearance. Qualitative studies with open‐ended questions could identify additional worries.

Future research should focus on determining the best method of identifying CCS in need of psychosocial support to cope with CRW. Therefore, the development and validation of measures with a clinical cut‐off for CRW are necessary. A promising measure is the Fear of Cancer Recurrence Inventory (FCRI), which has shown strong psychometric qualities, a proposed clinical cut‐off, and has been validated in survivors of both adult‐onset cancers and childhood cancers [60, 61]. Conversely, the Cancer Worry Scale (CWS) has been validated in adult CCS and addresses several worries, but no clinical cut‐off is available [52]. Distinguishing low levels from severe CRW is important as not all levels of CRW are problematic. Lower levels may promote adaptive behaviors such as adhering to screening and maintaining a healthy lifestyle [10], while clinical levels can lead to excessive healthcare use or avoidance of medical examinations, and may negatively impact quality of life [12, 14, 15, 16].

In this systematic review, several factors associated with increased CRW were identified. Future studies could explore additional factors, such as coping strategies, support needs, CRW in parents, partners and other family members, actual elevated cancer‐related risks, and triggers like bodily symptoms and medical examinations. Understanding these factors could help in developing targeted interventions to address CRW.

Although our review did not specifically address interventions for CRW among CCS and such studies are currently limited, it highlights the need for research into effective strategies for addressing it. Potential interventions could include providing information about late effects, psycho‐education, discussions during follow‐up care, and targeted CBT or ACT‐based interventions for severe CRW. Future research should evaluate the effectiveness of these approaches.

Lastly, there is little longitudinal research on the course of CRW in CCS over time. Future research could explore the progression of various CRW over time, spanning from treatment into long‐term survivorship. This provides insights into how these worries evolve and can further inform support strategies for CCS.

## 5 Conclusion

CRW are prevalent among CCS, particularly health‐related worries about future health and late effects, cancer recurrence and second cancers, and infertility. Factors associated with several specific increased CRW include female sex, more pain (intensity, interference, and information needs about pain), the presence of anxiety, depression and chronic conditions (being overweight or having a neurologic disorder), and a history of chemotherapy or (higher doses of) radiotherapy. Given the negative impact of CRW on the mental and physical health of CCS, it is crucial to identify CCS with severe CRW, for example by using validated measures. Addressing CRW through information provision, psycho‐education, follow‐up care discussions, and targeted psychosocial interventions for severe CRW could promote adaptive healthcare use and enhance the overall quality of life of CCS.

## Author Contributions

**Anne Maas:** conceptualization, methodology and search strategy, literature search and screening studies for inclusion, data extraction, quality assessment, writing–original draft. **Anne Westerweel:** conceptualization, methodology and search strategy, literature search and screening studies for inclusion, checking data extraction for accuracy, checking quality assessment for accuracy, writing–review & editing. **Heleen Maurice‐Stam:** conceptualization, methodology and search strategy, funding acquisition, writing–review & editing. **Leontien C. M. Kremer:** funding acquisition, writing–review & editing. **Alied M. van der Aa‐van Delden:** conceptualization, writing–review & editing. **Daniël Zwerus:** conceptualization, Writing–review & editing. **Elvira C. van Dalen:** conceptualization, methodology and search strategy, third‐party arbitrator in: screening studies for inclusion, data extraction and quality assessment, writing–review & editing. **Martha A. Grootenhuis:** conceptualization, funding acquisition, writing–review & editing.

## Conflicts of Interest

The authors declare no conflicts of interest.

## Supporting information

Supplementary Material

Table S4

## Data Availability

The protocol and the data that support the study findings are available upon reasonable request from the corresponding author.
