# Supplementary material for: The Prevalence and Associated Factors of Cancer‐Related Worries in Adult Survivors of Childhood Cancer: A Systematic Review
Source: Psychooncology. 2025 Feb 13;34(2):e70101. doi: 10.1002/pon.70101 (PMC11825232; doi:10.1002/pon.70101)
Supplement: Supplementary file 1 — Supplementary Material [file PON-34-e70101-s001.docx]

**Supplementary Table 1: Search strategy MEDLINE/PubMed**

| **1. Childhood cancer** | leukemia OR leukemi* OR leukaemi* OR "childhood ALL" OR AML OR (leukemia, lymphocytic, acute[mh]) OR (leukemia, lymphocytic, acute*) OR lymphoma OR lymphom* OR hodgkin OR hodgkin* OR T-cell OR B-cell OR non-hodgkin OR non-hodgkin* OR sarcoma OR sarcom* OR sarcoma, Ewing's OR Ewing* OR osteosarcoma OR osteosarcom* OR wilms tumor OR wilms* OR nephroblastom* OR neuroblastoma OR neuroblastom* OR rhabdomyosarcoma OR rhabdomyosarcom* OR teratoma OR teratom* OR hepatoma OR hepatom* OR hepatoblastoma OR hepatoblastom* OR PNET OR medulloblastoma OR medulloblastom* OR PNET* OR neuroectodermal tumors, primitive OR retinoblastoma OR retinoblastom* OR meningioma OR meningiom* OR glioma OR gliom* OR brain tumor OR brain tumor* OR brain tumour* OR brain cancer* OR brain neoplasm* OR intracranial neoplasm* OR brain neoplasms OR central nervous system neoplasm OR central nervous system neoplasms OR central nervous system neoplasm* OR central nervous system tumor OR central nervous system tumour OR central nervous system tumor* OR central nervous system tumour* OR pediatric oncology OR paediatric oncology OR childhood cancer OR childhood tumor OR childhood tumors OR childhood tumour OR childhood tumours OR childhood tumor* OR childhood tumour* |
| --- | --- |
| **2. Survivors** | Survivor OR survivors OR long term survivor OR long term survivors OR long term survivo* OR survivo* OR long term survival OR survival[mh] OR long-term survivor OR long-term survivors OR long-term survivo* OR childhood cancer survivor OR childhood cancer survivors OR childhood cancer survivo* OR cancer survivors[mh] |
| **3. Worries** | fear OR fears OR fear* OR worry OR worries OR worry* OR anxiety OR anxi* OR “angst” OR hypervigilance OR hypervigil* OR nervousness OR anxiousness OR stress, psychological[mh] |
| **Combination** | #1 AND #2 AND #3 |
| **Limitations** | English language |

**Supplementary Table 2: Search strategy PsycINFO**

| **1. Childhood cancer** | leukemia OR leukemi* OR leukaemi* OR "childhood ALL" OR AML OR (exp "leukemia, lymphocytic, acute"/) OR ("leukemia, lymphocytic, acute*" ) OR lymphoma OR lymphom* OR hodgkin OR hodgkin* OR T-cell OR B-cell OR non-hodgkin OR non-hodgkin* OR sarcoma OR sarcom* OR "sarcoma, Ewing's" OR Ewing* OR osteosarcoma OR osteosarcom* OR "wilms tumor" OR wilms* OR nephroblastom* OR neuroblastoma OR neuroblastom* OR rhabdomyosarcoma OR rhabdomyosarcom* OR teratoma OR teratom* OR hepatoma OR hepatom* OR hepatoblastoma OR hepatoblastom* OR PNET OR medulloblastoma OR medulloblastom* OR PNET* OR "neuroectodermal tumors, primitive" OR retinoblastoma OR retinoblastom* OR meningioma OR meningiom* OR glioma OR gliom* OR "brain tumor" OR "brain tumor*" OR "brain tumour*" OR "brain cancer*" OR "brain neoplasm*" OR "intracranial neoplasm*" OR "brain neoplasms" OR "central nervous system neoplasm" OR "central nervous system neoplasms" OR "central nervous system neoplasm*" OR "central nervous system tumor" OR "central nervous system tumour" OR "central nervous system tumor*" OR "central nervous system tumour*" OR "pediatric oncology" OR "paediatric oncology" OR "childhood cancer" OR "childhood tumor" OR "childhood tumors" OR "childhood tumour" OR "childhood tumours" OR "childhood tumor*" OR "childhood tumour*" |
| --- | --- |
| **2. Survivors** | Survivor OR survivors OR "long term survivor" OR "long term survivors" OR "long term survivo*" OR survivo* OR "long term survival" OR exp survival/ OR "long-term survivor" OR "long-term survivors" OR "long-term survivo*" OR "childhood cancer survivor" OR "childhood cancer survivors" OR "childhood cancer survivo*" OR exp "cancer survivors"/ |
| **3. Worries** | fear OR fears OR fear* OR worry OR worries OR worry* OR anxiety OR anxi* OR angst OR hypervigilance OR hypervigil* OR nervousness OR anxiousness OR exp "stress, psychological"/ |
| **Combination** | #1 AND #2 AND #3 |
| **Limitations** | English language |

*Note:* The search strategy for PubMed was adapted for APA PsycINFO using the Polyglot Search Translator [Clark JM, Sanders S, Carter M, Honeyman D, Cleo G, Auld Y, Booth D, Condron P, Dalais C, Bateup S, Linthwaite B, May N, Munn J, Ramsay L, Rickett K, Rutter C, Smith A, Sondergeld P, Wallin M, Jones M, Beller E. *Improving the translation of search strategies using the Polyglot Search Translator: a randomized controlled trial.* J Med Libr Assoc. 2020 Apr;108(2):195-207].

**Supplementary Table 3**: **Risk of bias assessment criteria for observational studies**

|  | **Internal validity** |  |
| --- | --- | --- |
| **Study group^#^** | **Selection Bias (is the study group representative?)**:  Low risk if:  The study group consisted of more than 75% of the original cohort of eligible participants  *or*  It was a random sample with respect to cancer treatment and important prognostic factors (attained age, sex). |  |
| **Follow-up^#^** | **Attrition bias (is the follow-up adequate?)**:  Low risk if:  The outcome was assessed for more than 75% of the study group |  |
| **Risk estimation^#/^**** | **Confounding (are the analyses adjusted for important confounders?)**:  Low risk if:  Important prognostic factors (attained age and sex) were taken adequately into account. |  |
| **Measurement bias** | **Measurement bias**  Low risk if:  Standardized, reliable, validated questionnaires were used |  |

*Note: Each bias item was scored as low risk, high risk or unclear risk (no overall scores were calculated); attrition bias and measurement bias were scored for each outcome separately*

*# From Cochrane Childhood Cancer risk of bias criteria for observational studies, based on previously described checklists according to evidence-based medicine criteria [Grimes, D.A. and K.F. Schulz, Cohort studies: marching towards outcomes. Lancet, 2002. 359(9303): p. 341-5; Laupacis A, Wells G, Richardson WS, Tugwell P. Users' guides to the medical literature. V. How to use an article about prognosis. Evidence-Based Medicine Working Group. JAMA. 1994 Jul 20;272(3):234-7. doi: 10.1001/jama.272.3.234]*

*** only applicable when risk factor analyses have been performed*
