# Supplementary material for: The Prevalence and Associated Factors of Cancer‐Related Worries in Adult Survivors of Childhood Cancer: A Systematic Review
Source: Psychooncology. 2025 Feb 13;34(2):e70101. doi: 10.1002/pon.70101 (PMC11825232; doi:10.1002/pon.70101)
Supplement: Supplementary file 2 — Table S4 [file PON-34-e70101-s002.docx]

| **Supplementary table 4: Included studies on cancer-related worries** | | | | | | | | |
| --- | --- | --- | --- | --- | --- | --- | --- | --- |
| **Study, country** | **Cancer-related worries** | **Study design** | **Sample survivors**  **(N, sex, age at evaluation (years), age at primary cancer diagnosis (years), follow-up (years), cancer type & treatment, treatment era)** | **Control group (if applicable)**  **(N/type, sex, age at evaluation (years))** | **Measures, Cronbach’s alpha (if applicable)** | **Results (prevalence, mean)** | **Results (associated factors)** | **Risk of bias assessment** |
| Balcerek, M., et al., *Nationwide Survey on the Health of Offspring from Former Childhood Cancer Patients in Germany.* Klin Padiatr, 2015. **227**(6-7): p. 350-4.  Country: Germany | Worries about offspring | Cross-sectional | N: 254 CCS who have one or more children born after their cancer treatment answered questions about their child(ren) (n = 418)  Sex: 69.3% female  Age at evaluation: Mean, SD: not reported, (range 20-43), Median 31  Age at primary cancer diagnosis: Mean, SD: not reported, (range 0-14), Median 10  Follow-up: Time since diagnosis: not reported. Based on the range of age at diagnosis and age at time of survey, it follows that CCS are ≥ 6 years from diagnosis  Cancer type: Mixed, Leukemia most common (64.0%)  Treatment type: not reported | No control group | Worries about offspring  A questionnaire based on the Robert Koch Institute’s health survey (KiGGS study) on children’s health in the German general population. Fear was assessed with two items: ‘How anxious are you that your child may develop cancer?’, and ‘Are you worried that your child may develop (further) health problems in the future?’ CCS could mark their fear on a 10cm long visual analog scale from 0 (no anxiety) to 10 (very high anxiety). | Anxiety of possible cancer development in offspring  *N=336 children for whom answers were given*  No to very low anxiety (0-20.0mm): 26.5%;  Low anxiety (20.1-40.0mm): 17.3%;  Moderate anxiety (40.1-60.0mm): 20.8%;  High anxiety (60.1-80.0mm): 16.1%;  Very high anxiety (80.1-100.0mm): 19.3%  Worry of development of (further) health problems in the future in offspring  *N=391 children for whom answers were given*  Yes: 19.9%;  No: 80.1% | Not reported | A. Selection bias  **Unclear risk**  Reason: The original cohort of eligible survivors is unknown. It should be noted that females, former patients with highest educational levels, and former patients who had survived soft tissues tumors were more likely to respond to the survey.  B. Attrition bias  **Low risk**  Reason: There is an outcome assessment for at least 336 out of 418 (80.4%) children for whom answers were given.  C. Confounding  Not applicable, associated factors not analyzed.  D. Measurement bias  **High risk**  Reason: Worries were measured with two single items that were not standardized or validated. |
| Berg, C.J., et al., *Young Adult Cancer Survivors' Experience with Cancer Treatment and Follow-Up Care and Perceptions of Barriers to Engaging in Recommended Care.* J Cancer Educ, 2016. **31**(3): p. 430-42.  Country: USA | Fear of cancer recurrence | Cross-sectional | N: 106 survivors  Sex: 50.0% female  Age at evaluation: Mean 22.14 SD 3.16 (range 18-34)  Age at primary cancer diagnosis: Mean and SD unknown (<18)  Follow-up: Time since diagnosis: Mean 8.4 SD 5.7  Cancer type: Mixed, Hodgkin’s lymphoma most common (22.6%)  Treatment type:  Chemotherapy (81.1%); Surgery (77.4%); Radiotherapy (55.7%)  Treatment era: not reported | No control group | Fear of cancer recurrence  Assessed with a single item: ‘Do you worry that your cancer will come back?’. Responses on a 5-point Likert scale from 0 (almost never) to 4 (almost always). | Fear of cancer recurrence  Total: Mean 1.65 SD 1.36 | Not reported. | A. Selection bias  **Unclear risk**  Reason: The original cohort of eligible survivors is unknown.  B. Attrition bias  **Unclear risk**  Reason: it is unclear how many participants have an outcome assessment on fear of cancer recurrence. It is only stated that the total is not equaling 106 due to missing data.  C. Confounding  Not applicable, associated factors not analyzed.  D. Measurement bias  **High risk**  Reason: fear of cancer recurrence is assessed with a single item that was not standardized or validated. |
| Cherven, B., et al., *Fertility-related worry among emerging adult cancer survivors.* J Assist Reprod Genet, 2022. **39**(12): p. 2857-2864.  Country: USA | Worry about infertility | Cross-sectional | N: 249 cancer survivors with a history of gonadotoxic treatment  Sex: 44.2% female  Age at evaluation: Mean 19.1 SD 1.2 (range 18.0-25.3), Median 18.7  Age at primary cancer diagnosis: Mean 9.6 SD 5.6 (range 0.1-21.9), Median 10.3  Follow-up: Time from cancer treatment completion: Mean 8.0 SD 5.2 (range 1.0-21.5), Median 6.7  Cancer type: Leukemia (36.5%); Lymphoma (26.1%); Solid tumor (37.3%)  Treatment type: Gonadotoxic therapies: Chemotherapy, Radiotherapy and/or Hematopoietic cell transplant  Treatment era: not reported | No control group | Worry about infertility  Structured infertility-focused discussions which assessed worry about future infertility (yes/no) through a direct question, or through expressed worry by the survivor in the consult. These data were abstracted from the medical record.  Treatment-related risk for infertility (low, moderate, or high risk) was determined based on each survivor’s gonadotoxic exposures, using the risk stratification consensus available at the time of patient counseling.  Fertility discussions: number of fertility discussions in survivorship care. | Worry about infertility per group:  Total: 66.3%;  Females: 75.5%;  Males: 59.0%  Leukemia survivors: 53.8%;  Lymphoma survivors: 66.2%;  Solid tumor survivors: 78.5%  Low infertility risk: 56.3%;  Moderate infertility risk: 76.9%;  High infertility risk: 80.9%  1 infertility discussion: 58.3%;  ≥2 fertility-focused discussions: 79.6% | Multivariable analysis  Significant factors related to fertility-related worry:  Female vs male sex: OR: 2.64 (95% CI: 1.44–4.96, p: 0.002)  Solid tumor diagnosis vs leukemia: OR: 2.31 (95% CI: 1.15–4.71, p: 0.019)  Moderate risk of infertility vs low risk: OR: 2.94 (95% CI: 1.23–7.64, p: 0.02)  High risk of infertility vs low risk: OR: 3.25 (95% CI: 1.55–7.17, p: 0.002)  ≥2 vs 1 fertility discussions during survivorship care: OR: 2.71 (95% CI: 1.46–5.20, p: 0.002)  Not significant  *Multivariable*  Lymphoma vs leukemia. Using backwards elimination, time from cancer treatment completion and hormonal laboratory evaluation were eliminated from the model.  *Univariable*  Age at first fertility discussion during survivorship, age at diagnosis, time from cancer treatment completion, race/ethnicity, religion, geographical location by rural status, insurance type, fertility preservation consult and/or procedure before treatment. | A. Selection bias  **Unclear risk**  Reason: The original cohort of eligible survivors is unknown.  B. Attrition bias  **Low risk**  Reason: All participants (100%) had documentation of fertility-related worry.  C. Confounding  **High risk**  Reason: Sex was included in the multivariable analysis, but age at evaluation was not. Univariable analyses were also high risk.  D. Measurement bias  **High risk**  Reason: The measure for fertility-related worries relies on a single question that is not standardized or validated. Alternatively, the outcome was based on whether the worry was expressed during the consultation. |
| Cox, C.L., et al., *Promoting physical activity in childhood cancer survivors: results from the Childhood Cancer Survivor Study.* Cancer, 2009. **115**(3): p. 642-54.  Country: USA | Fear about future health  Fear of cancer recurrence  Fear of the discovery of a health problem at check-up | Cross- sectional | Total N: 838 Structural Equation Modeling (SEM) subsamples, Men N: 256, Women N: 363  Sex: 54.1% female  Age at evaluation: Mean 30.98 SD 7.50 (range: not reported, adult CCS)  Age at primary cancer diagnosis: Mean 9.25 SD 5.87 (< 21)  Follow-up: Time since diagnosis: Mean 21.74 SD 4.54 (≥ 5 after treatment)  Cancer type: Leukemia, Hodgkin disease, Lymphoma (66.7%); Solid tumor (23.1%); Bone cancer (10.2%)  Treatment type: not reported  Treatment era: CCS diagnosed between 1970 and 1986 | No control group | Cancer-related fears Assessed with 3 items about the extent of the fear of CCS about their future health, the recurrence of their cancer, and the discovery of a health problem during a routine check-up. Responses on a 5-point Likert scale from 1 (not at all concerned) to 5 (extremely concerned). Cronbach’s α: 0.76. | Mean item scores, and comparison with SEM subsamples  Fear about future health  Total: Mean 2.86 SD 1.08  SEM subsamples: Men, Mean 2.75 SD 1.09. Women, Mean 2.95 SD 1.04  Fear of cancer recurrence  Total: Mean 2.39 SD 1.17  SEM subsamples: Men, Mean 2.29 SD 1.13.Women, Mean 2.50 SD 1.19  Fear of the discovery of a health problem at check-up  Total: Mean 2.23 SD 1.16  SEM subsamples: Men, Mean 2.07 SD 1.07. Women, Mean 2.39 SD 1.22 | Univariable analysis  Females vs males  Fear about future health  Differences between SEM subsamples not significant.  Fear of cancer recurrence  Differences between SEM subsamples not significant.  Fear of the discovery of a health problem at check-up  Subsample women more likely to be fearful of finding a problem at check-up than subsample men. F-ratio, p: 0.003; Post Hoc, p: 0.001 | A. Selection bias  **High risk**  Reason: The original cohort of eligible survivors consisted of 20,346 CCS of which 12,872 were alive at time of study, and 838 participated (6.5%).  B. Attrition bias  **Low risk for the total group and for the SEM subsample of women, high risk for the SEM subsample of men**  Reason: There is an assessment on cancer-related fears for at least 832 out of 838 (99.3%) participants of the total group. For the SEM subsamples, there is an outcome assessment for 363 (80.1%) of women, and for 256 (66.5%) of men.  C. Confounding  **High risk**  Reason: Univariable analysis.  D. Measurement bias  **High risk**  Reason: Each cancer-related fear was measured with a single item that was not standardized or validated. |
| Cox, C.L., et al., *The unmet emotional, care/support, and informational needs of adult survivors of pediatric malignancies.* J Cancer Surviv, 2016. **10**(4): p. 743-58.  Country: USA | Concern about ability of having children  Concern about developing cancer in the future  Concern about ability to get health insurance | Cross-sectional | N: 1,189 survivors, excluding those treated at St. Jude Children’s Research Hospital  Sex: 60.9% female  Age at evaluation: Mean 39.7 SD 7.7 (range 26-61)  Age at primary cancer diagnosis: Mean, SD: not reported, (range 0-20): 0-4 (38.0%);  5-9 (22.8%); 10-14 (20.8%);  15-20 (18.4%)  Follow-up: Time since diagnose: Mean 31.6 SD 4.7 (range 24-42)  Cancer type: Mixed, Leukemia most common (33.7%)  Treatment type:  Chemotherapy (76.5%); Radiotherapy (65.0%); Surgery (80.0%)  Treatment era: diagnosed between 1970 and 1986 | No control group | Cancer-related worries Assessed with 3 single items: ‘Please rate how concerned you are about the following: 1) your ability to have children; 2) developing cancer in the future; 3) your ability to get health insurance’. Responses on a 5-point scale from 1 (not at all concerned) to 5 (very concerned).  ‘Not at all/not very much’ and ‘Somewhat/very much’, and ‘None/not very’ and ‘Somewhat/very’ were not further specified. | Concern about ability of having children: Not at all/not very much: 73.4%;  Somewhat/very much: 26.6%  Concern about developing cancer in the future: None/not very: 39.6%;  Somewhat/very: 60.4%  Concern about ability to get health insurance: Not at all/not very much: 64.2%;  Somewhat/very much: 35.8% | Not reported for cancer-related worries as outcomes | A. Selection bias  **High risk**  Reason: At least 4,454 CCS were eligible, and 1,189 CCS completed the questionnaires (26.7%).  B. Attrition bias  **Low risk**  Reason: There is an assessment on cancer-related worries for 1,179 out of 1,189 participants (99.2%).  C. Confounding  Not applicable, associated factors not analyzed.  D. Measurement bias  **High risk**  Reason: cancer-related worries are assessed with 3 single items that were not standardized or validated. |
| Dalkner, N., et al., *Parenthood for childhood cancer survivors: unfounded fear of cancer development in offspring and related health behaviors.* Front Psychol, 2023. **14**: 1269216.  Country: Austria, Czech Republic, Germany, Poland, Switzerland | Worries about offspring | Cross-sectional | N: 256 CCS who have one or more children born after their cancer treatment  Sex: 66.0% female  Age at evaluation Mean 38.5 SD 6.0 (≥ 18)  Age at primary cancer diagnosis Mean 10.42 SD 4.48 (range not reported, diagnosis during childhood)  Follow-up: Time since diagnosis Mean 28.10 SD 6.10 (range not reported)  Cancer type: Mixed, Leukemia most common (44.9%)  Treatment type: not reported | N/type: 256 age- and sex- matched siblings  Sex: 66% female  Age at evaluation: Mean 38.7 SD 6.6 | Worries about offspring  A questionnaire based on the Robert Koch Institute’s health survey (KiGGS study) on children’s health was distributed in Austria, the Czech Republic, Germany, Poland, and Switzerland. Fear was assessed with the item: ‘How worried are you that your child might develop cancer?’ by marking their fear on a 10cm long visual scale form 0 (little to no fear) and 10 (highly fearful). | Fear of possible cancer development in offspring  CCS:  Mean: 4.10 SD 2.96  None/low (<2cm): 36.7%;  Medium (2-6cm): 36.7%;  High/very high (>6 cm): 26.6%  Siblings:  Mean: 3.52 SD 2.84  None/low: 42.6%;  Medium: 36.3%;  High/very high: 21.1%  CCS had higher levels of fear of possible cancer development in offspring than siblings, adjusted for age and sex of parent and child, country, and highest vocational education in household (p: 0.044). | Multivariable analyses (partial correlation)  adjusted for sex of parent and child, age of child, country, highest vocational education in household, type of cancer, age when treated. Analysis for time since therapy and number of children were also adjusted for age of parent.  Significant association with fear of possible cancer development in offspring (CCS)  Younger age of parent (current): r: -0.16, p: 0.014  Shorter time since therapy: r: -0.19, p: 0.003  Lower number of children: r: -0.21, p: 0.001  Not significant (after the employment of false discovery rate) Age of parent at oncological therapy, age of child (current), number of doctors’ appointments in the last 12 months, number of total vaccinations, risk behavior score. | A. Selection bias  **Unclear risk**  Reason: The original cohort of eligible survivors is unknown.  B. Attrition bias  **Low risk**  Reason: There is an outcome assessment for all 256 CCS and siblings (100%).  C. Confounding  **Low risk**  There is accounted for age at evaluation and sex of parent and child in the analysis.  D. Measurement bias  **High risk**  Reason: Fears were measured with one single item that was not standardized or validated. |
| Fair, D., et al., *Material, behavioral, and psychological financial hardship among survivors of childhood cancer in the Childhood Cancer Survivor Study.* Cancer, 2021. **127**(17): p. 3214-3222.  Country: USA | Worries about finances  Worries about insurance coverage | Cross-sectional | N: 698 of which 10.2% (n: 79) uninsured  Sex: 54.5% female  Age at evaluation: Mean, SD: not reported  22-29 (11.3%) 30-39 (42.3%) ≥40 (46.4%)  Age at primary cancer diagnosis: Mean, SD: not reported  0-5 (46.4%) 6-10 (19.1%) 11-15 (19.8%) 16-20 (14.7%)  Follow-up: Time since diagnosis: Mean not reported, SD not reported (range 23.1-41.7), Median 28.8  Cancer type: Mixed, Leukemia most common (35.0%)  Treatment type: Chemotherapy (76.7%); Radiotherapy (65.6%); Surgery (80.0%); Neither chemotherapy, radiotherapy, surgery (8.1%)  Treatment era: diagnosed between 1970 and 1986 | N/type: 210 siblings of which 7.9% (n: 21) uninsured  Sex: 61.1% female  Age at evaluation Mean, SD: not reported  22-29: 13.5% 30-39: 33.6% ≥40: 52.9% | Psychological financial hardship  4 items adapted from the National Health Interview Survey about worries e.g., losing their jobs or being unable to pay medical bills in the past year. Other items not reported.  Insurance worries  4 items for insured participants regarding worries about current insurance coverage e.g., concern their health insurance will become so expensive they wouldn’t be able to afford it. Other items not reported. | Psychological financial hardship  CCS:  No instances of worries: 56.8% ^e^;  Instances of worries ^d^: 43.2% (1-2 instances: 26.5%; ≥ 3 instances: 16.7%)  Siblings:  No instances of worries: 64.1%  Instances of worries ^d^: 35.9% (1-2 instances: 23.0%; ≥ 3 instances: 12.9%)  Proportions of psychological financial hardship did not significantly differ between CCS and siblings (p: 0.07), adjusted for insurance.  Psychological worries related to insurance coverage (insured participants only)  CCS:  No instances of worries: 45.5%;  Instances of worries ^d:^ 54.5% (1-2 instances: 22.8%; ≥ 3 instances: 31.7%)  Siblings:  No instances of worries: 49.9%;  Instances of worries ^d^: 50%  (1-2 instances: 22.1%; ≥ 3 instances: 27.9%)  Among insured, psychological worries regarding insurance coverage did not differ between CCS and siblings p: 0.28). | Multivariable analyses Including insured status, age at diagnosis (0-4 vs 5-20 years), years since diagnosis, sex (21-29 vs ≥30 years), non-Hispanic white ethnicity, presence of any chronic condition, marital status (married vs not married), and diagnosis.  Significant factors associated with psychological financial hardship (≥3 vs 0-2 instances):  Insured status (vs non-insured): OR: 0.10 (95% CI: 0.05-0.21, p < 0.001)  Younger age (0-4 years) at diagnosis vs 5-20 years: OR: 0.40 (95% CI: 0.22-0.75, p: 0.004)  Female vs male sex: OR: 2.19 (95% CI: 1.30-3.70, p: 0.003)  Non-Hispanic white ethnicity vs other: OR: 0.40 (95% CI: 0.17-0.92, p: 0.03)  Diagnosis leukemia vs bone tumor: OR: 3.12 (95% CI: 1.05-9.27, p: 0.04)  Not significant  Years since diagnosis, presence of any chronic condition, marital status, other diagnosis than leukemia.  Significant factors associated with psychological worries related to insurance coverage (≥3 vs 0-2), insured only:  Younger age (0-4 years) at diagnosis (vs 5-20 years): OR: 0.57 (95% CI: 0.35-0.92, p: 0.02)  ≥30 years since diagnosis (vs 21-29 years): OR: 1.68 (95% CI: 1.12-2.50, p: 0.01)  Female vs male sex: OR: 1.93 (95% CI: 1.29-2.89, p: 0.002)  Not significant  Non-Hispanic white ethnicity, presence of any chronic condition, marital status, and diagnosis. | A. Selection bias  **High risk**  Reason: 14,357 CCS were eligible, and 698 CCS completed the questionnaires (4.9%).  B. Attrition bias  **Low risk**  Reason: Of the 698 CCS, 677 (97.0%) had an outcome assessment on psychological financial hardship, and 605 (96.5%) out of the 627 insured CCS had an outcome assessment on insurance worries.  C. Confounding  **Low risk**  Reason: Sex was included in the multivariable analysis, and the samples were age-stratified.  D. Measurement bias  **Unclear risk**  Reason: The measure was adapted from the National Health Interview Survey and additional items were added. It is unclear whether the adjusted measure is validated. |
| Fisher, A.P., et al., *Piloting a Survivorship Screening Tool in a Specialty Clinic for Survivors of Childhood Cancers.* J Adolesc Young Adult Oncol, 2020. **9**(3): p. 418-421.  Country: USA | Fear of cancer recurrence | Cross-sectional | N: 114 CCS visiting the survivorship clinic  Sex: not reported  Age at evaluation Mean 33.5 SD 7.7 (range 20-56)  Age at primary cancer diagnosis: Mean, SD, range: not reported (diagnosis in childhood)  Follow-up: Time since diagnosis: Mean 23.8 SD 8.1 (range not reported)  Cancer type: Mixed, Leukemia most common (29%)  Treatment type: Chemotherapy (95%); Radiotherapy (75%); Surgery (38%);  Transplant (22%)  Treatment era: not reported | No control group | Just before or during their annual appointment in the survivorship clinic, CCS completed a screening tool of 42 items including:  Fear of recurrence  A single item assessing fear of cancer recurrence in the past 7 days. Response options: ‘not at all’, ‘several days’, ‘more than half the days’, and ‘nearly every day’. As outcome in the regression analysis these categories were collapsed into ‘fear’ and ‘no fear’. These categories were not further specified.  Survivorship concerns  CCS could select out of a total of 32 physical, psychosocial, and practical concerns. | Fear of recurrence in the past week  Not at all: 75%;  Any number of days ^d^: 25% (Several days: 18%; More than half the days: 7%; Nearly every day: 0%) | Univariable analysis  Significant association with fear of recurrence (fear vs no fear)  Higher number of survivorship concerns: OR: 1.4 (95% CI: 1.2-1.6, p < 0.0001)  Not significant  Number of late effects, age at diagnosis, secondary malignancy, previous recurrence. | A. Selection bias  **Unclear risk**  Reason: The original cohort of eligible survivors is unknown.  B. Attrition bias  **Low risk**  Reason: There is an assessment on fear of recurrence for 100 out of 114 (87.7%) participants.  C. Confounding  **High risk**  Reason: Univariable analysis, so there is not accounted for age at evaluation and sex in the analysis.  D. Measurement bias  **High risk**  Reason: Fear of recurrence was measured with a single item that was not standardized or validated. |
| Ford, J.S., et al., *Psychosocial Outcomes in Adult Survivors of Retinoblastoma.* J Clin Oncol, 2015. **33**(31): p. 3608-14.  Country: USA | Fear of cancer recurrence  Concern about future health  Concern about ability to have children  Concern about developing another cancer  Worry about possibility of children getting retinoblastoma (RB) | Cross-sectional | N: 470 retinoblastoma (RB) survivors treated in New York  Sex: 52.1% female  Age at evaluation: Mean 43.3 SD 11 (≥ 18)  Age at primary cancer diagnosis: Mean, SD: not reported, (range 0-17), Median 1 years  Follow-up: Time since diagnosis: Mean, SD, range: not reported.  Cancer type: Retinoblastoma, unilateral (46.4%) and bilateral (53.6%)  Treatment type: Chemotherapy (25.3%); Surgery (86.0)%; Radiotherapy (56.4%)  Treatment era: CCS diagnosed between 1932 and 1994 | No control group for cancer-related worries | Fear of cancer recurrence  Assessed using the Fear of Recurrence Questionnaire, consisting of 22 items, like ‘I think about my health often’, ‘I do not worry about my illness returning’, ‘When I think about my future health status, I feel some uneasiness’. Responses on a 5-point Likert scale from 1 (strongly agree) to 5 (strongly disagree). Higher scores indicate higher fear. Cronbach’s α: 0.92.  Concern about future health  Assessed with the item ‘Please rate how concerned you are about the following: your future health’. Responses on a 5-point Likert scale from 1 (not at all concerned) to 5 (very concerned).  Concern about ability to have children Assessed with the item ‘Please rate how concerned you are about the following: your ability to have children’. Responses on a 5-point Likert scale from 1 (not at all concerned) to 5 (very concerned).  Concern about developing another cancer  Assessed with the item ‘Please rate how concerned you are about the following: developing a cancer’. Responses on a 5-point Likert scale from 1 (not at all concerned) to 5 (very concerned).  Worry about possibility of children getting RB: not reported  Never, rarely, sometimes and often were not further specified. | Fear of cancer recurrence  Survivors of unilateral RB: Mean 42.8 SD 10.6  Survivors of bilateral RB: Mean 50.2 SD 10.8  Concern about future health  Total:  Not at all concerned: 7.9%;  Concerned ^d:^ 92.1%;(Somewhat/concerned/not very: 76.6%; Very concerned: 15.5%)  Unilateral:  Not at all concerned: 13.2%;  Concerned ^d^: 86.7% (Somewhat/concerned/not very: 77.7%; Very concerned: 9.0%)  Bilateral:  Not at all concerned: 3.2%;  Concerned ^d^: 96.8% (Somewhat/concerned/not very: 75.7%; Very concerned: 21.1%)  Concern about ability to have children  Total:  Not at all concerned: 66.4%;  Concerned ^d^: 33.7% (Somewhat/concerned/not very: 24.5%; Very concerned: 9.2%)  Unilateral:  Not at all concerned: 70.1%;  Concerned ^d^: 29.9% (Somewhat/concerned/not very: 22.3%; Very concerned: 7.6%)  Bilateral:  Not at all concerned: 63.1% ^f^;  Concerned ^d :^ 36.9% (Somewhat/concerned/not very: 26.2%; Very concerned: 10.7%)  Concern about developing another cancer  Total:  Not at all concerned: 9.4%;  Concerned ^d :^ 90.6%  (Somewhat/concerned/not very: 70.5%; Very concerned: 20.1%)  Unilateral:  Not at all concerned: 15.6%;  Concerned ^d :^ 84.4%  (Somewhat/concerned/not very: 73.0%; Very concerned: 11.4%)  Bilateral:  Not at all concerned: 4.1%;  Concerned ^d^: 94.1% (Somewhat/concerned/not very: 68.4%; Very concerned: 27.5% ^g^)  Worry about possibility of children getting RB  Total:  Never/rarely: 47.7%;  Sometimes/often: 52.3%  Unilateral:  Never/rarely: 55.9%;  Sometimes/often: 44.1%  Bilateral:  Never/rarely: 40.7%;  Sometimes/often: 59.3% | Univariable analysis  Fear of cancer recurrence  Survivors of unilateral RB had significantly lower mean scores compared with survivors of bilateral RB (p: < 0.01).  Concern about future health  Significant difference in the proportions of concern between unilateral and bilateral CCS (p < 0.01), with bilateral CCS being more concerned.  Concern about ability to have children  No significant difference in the proportions of concern between unilateral and bilateral CCS.  Concern about developing another cancer  Significant difference in the proportions of concern between unilateral and bilateral CCS (p < 0.01), with bilateral CCS being more concerned.  Worry about possibility of children getting RB  Significant difference in the proportions of concern between unilateral and bilateral CCS (p < 0.01), with bilateral CCS being more concerned. | A. Selection bias  **Unclear risk**  Reason: The original cohort of eligible survivors is unknown.  B. Attrition bias  **Low risk**  Reason: There is an assessment on cancer-related worries for at least 89.4% of the participants.  C. Confounding  **High risk**  Reason: Univariable analysis.  D. Measurement bias  **Unclear risk**  Reason: The Fear of Recurrence Questionnaire is validated and has an acceptable Cronbach’s α. However, it is unclear whether the questions about the other fears are validated. |
| Gibson, T.M., et al., *Perceptions of future health and cancer risk in adult survivors of childhood cancer: A report from the Childhood Cancer Survivor Study.* Cancer, 2018. **124**(16): p. 3436-3444.  Country: USA | Concern about future health  Concern about developing cancer | Cross-sectional ^b^ | N: 15,620 CCS  Sex: 48.1% female  Age at evaluation: Mean, SD: not reported, Median 26 (range 22-31)  Age at primary cancer diagnosis: Mean, SD: not reported. Median 8 (range: 4-13)  Follow-up: Time since diagnosis: Mean, SD: not reported. Median 17 (range 14-21)  Cancer type: Mixed, Leukemia most common (37.2%)  Treatment type: No surgery, radiation, or chemotherapy: 0.2%  Radiation and chemotherapy, no surgery: 11.1%  Surgery only: 7.6%  Radiation only: 0.3%  Chemotherapy only: 15.8%  Radiation and surgery, no chemotherapy: 8.3%  Chemotherapy and surgery, no radiation: 21.9%  Radiation, chemotherapy and surgery: 35.0%  Treatment era: CCS diagnosed between 1970 and 1999. | N/type: 3,991 siblings  Sex: 53.9% female  Age at evaluation: Mean, SD: not reported, Median 29 (range 24-35) | Participants rated how concerned they were about 1) their future health and 2) developing a cancer. Responses on a 5-point Likert scale from very concerned to not at all concerned. | Concern About Future Health  CCS:  Not at all concerned: 12.0%;  Concerned ^d^: 88.1% (Not very concerned: 18.7%;  Concerned: 23.2%; Somewhat concerned: 21.4%; Very concerned: 24.8%)  Siblings:  Not at all concerned: 12.0%;  Concerned ^d^: 88% (Not very concerned: 21.6%;  Concerned: 24.1%; Somewhat concerned: 22.2%; Very concerned: 20.1%)  The prevalence of concern about future health was higher in CCS compared to their siblings: RR: 1.12 (95% CI: 1.09-1.15)  Concern About Developing Cancer  CCS:  Not at all concerned: 17.2%;  Concerned ^d:^ 82.8% (Not very concerned: 22.8%;  Concerned: 21.1%; Somewhat concerned: 18.3%; Very concerned: 20.6%)  Siblings:  Not at all concerned: 16.3%;  Concerned ^d:^ 83.8% (Not very concerned: 22.2%;  Concerned: 25.1%; Somewhat concerned: 18.4%; Very concerned: 18.1%)  The prevalence of concern about developing cancer was similar compared to their siblings: RR: 1.02 (95% CI, 0.99-1.05). | Multivariable analysis adjusted for age, sex, race/ethnicity, year of diagnosis, education and prior grade 3 or 4 chronic conditions (concern about future health) or subsequent neoplasms (concerns about developing cancer).  Significant factors associated to prevalence of concern about future health  <20 Gy radiation dose, location not reported (vs none): RR: 1.07 (95% CI: 1.02-1.11);  ≥20 Gy radiation dose, location not reported (vs none): RR: 1.13 (95% CI: 1.09-1.16)  Chest radiation (yes vs no): RR: 1.08 (95% CI: 1.05-1.10)  Neck radiation (yes vs no): RR: 1.08 (95% CI: 1.05-1.11)  Chemotherapy:  Alkylating agent (vs no alkylating agent): RR: 1.07 (95% CI: 1.04-1.10);  Anthracyclines (vs no anthracyclines): RR: 1.06 (95% CI: 1.03-1.09)  Not significant  Cranial radiation, chemotherapy: platinum compounds  Significant factors associated to prevalence of concern about developing cancer  ≥20 Gy radiation dose, location not reported (vs none): RR: 1.14 (95% CI: 1.10-1.18)  Chest radiation (yes vs no): RR: 1.09 (95% CI: 1.05-1.12)  Neck radiation (yes vs no): RR: 1.09 (95% CI: 1.05-1.12)  Chemotherapy:  Alkylating agent (vs no alkylating agent): RR: 1.05 (95% CI: 1.01-1.08);  Anthracyclines (vs no anthracyclines): RR: 1.06 (95% CI: 1.02-1.09)  Not significant  <20 Gy radiation dose (location not reported), cranial radiation, chemotherapy: platinum compounds | A. Selection bias  **Unclear risk**  Reason: The original cohort of eligible survivors is unknown.  B. Attrition bias  **Low risk**  Reason: at least 99.4% (15,528) of participants had an outcome assessment.  C. Confounding  **Low risk**  Reason: There is accounted for age at evaluation and sex in the analysis.  D. Measurement bias  **High risk**  Reason: concerns about future health and developing another cancer were both assessed with a single item that was not standardized or validated. |
| Kelada, L., et al., *Perceived cancer-related pain and fatigue, information needs, and fear of cancer recurrence among adult survivors of childhood cancer.* Patient Educ Couns, 2019. **102**(12): p. 2270-2278.  Country: Australia | Fear of cancer recurrence | Cross-sectional | N: 404  Sex: 56.4% female  Age at evaluation: Mean 26.27 SD 7.65 (range 16-61)  Age at primary cancer diagnosis: Mean, SD: not reported (<16 years)  Follow-up: Time since diagnosis: Mean 19.05 SD 8.08 (range 5-59)  Cancer type: Mixed, Leukemia most common (42.6%)  Treatment type: Chemotherapy (89.6%); Radiotherapy (47.0%); Surgery (45.8%); Bone marrow transplant (17.3%)  Treatment era: not reported | No control group | Fear of Cancer Recurrence  A shortened version of the severity subscale of the Fear of Cancer Recurrence Inventory, 4 items. CCS could answer on a scale from 1 (not at all) to 5 (a great deal), with higher scores associated with more fear. Cronbach’s α: 0.84.  Pain and fatigue  Two checkbox items assessing health conditions including pain (options: 'previous pain', and 'anticipated pain'), and fatigue (options: 'previous fatigue', and 'anticipated fatigue'). Participants could select more than one option.  Information needs about managing pain and fatigue  Two questions were asked to assess the need of information about managing pain and fatigue: ‘since finishing  cancer treatment, have you had a need for information about the following?: 1) managing pain; 2) managing fatigue’. Response options: 1 (not needed, reference group); 2 (needed but not received); 3 (needed and received some); 4 (needed and received enough). The answers were coded into: ‘not needed’ = 1; ‘unmet needs’ = 2 and 3; ‘met needs’ = 4. | Fear of cancer recurrence  Mean 2.26 SD 0.83;  (range 1-5)  No fear of cancer recurrence: 11%;  Fear of cancer recurrence: 87.7% ^d^ (‘A little’ or ‘some’ fear of cancer recurrence: 79.0%; ‘A lot’ or ‘a great deal‘ of fear of cancer recurrence: 8.7%) | Univariable analysis  Significant associations with fear of cancer recurrence  Older age at study participation: r: 0.11, p: 0.024  More pain: r: 0.13, p: 0.010  More anticipated pain: r: 0.19, p < 0.001  More fatigue: r: 0.18, p: 0.001  More anticipated fatigue: r: 0.21, p: < 0.001  More unmet needs for information about pain: r: 0.34, p < 0.001  More unmet needs for information about fatigue: r: 0.32, p < 0.001  Female (vs male): r: 0.17, p: 0.001  Married status (vs unmarried): r: -0.11, p: 0.026  Lymphoma diagnosis (vs all other diagnoses combined): r: 0.14, p: 0.004  Relapse during childhood: r: 0.11, p: 0.026  Not significant  Years since diagnosis, years since treatment completion, met needs for information about pain and fatigue, ethnic background, education, employment, household income AUD, other diagnosis than lymphoma, treatment, currently attend long-term follow-up clinic.  Multivariable analysis including all variables that were significantly associated to fear of cancer recurrence univariably and including the variables about the met needs for information about pain and fatigue.  Significant associations with fear of cancer recurrence  More unmet needs for information about pain: B: 0.48 (95% CI: 0.19 – 0.76, p: 0.001)  More unmet needs for information about fatigue: B: 0.29 (95% CI: 0.06 – 0.52, p: 0.015)  Female (vs male): B: 0.18 (95% CI: 0.01 – 0.35, p: 0.035)  Lymphoma diagnosis (vs all other diagnoses combined): B: 0.29 (95% CI: 0.04 – 0.53, p: 0.021)  Not significant  Age at study participation, pain, anticipated pain, fatigue, anticipated fatigue, met needs for information about pain and fatigue, married status, relapse during childhood. | A. Selection bias  **Unclear risk**  Reason: The original cohort of eligible survivors is unknown. It should be noted that respondents were more likely to be female, older and be survivors of lymphoma than non-respondents.  B. Attrition bias  **Low risk**  Reason: There is an outcome assessment for at least 398 out of 404 (98.5%) participants.  C. Confounding  **Low risk for multivariable analysis, high risk for univariable analyses.**  Reason: There is accounted for age at evaluation and sex in the multivariable analysis, but not in the univariable analyses.  D. Measurement bias  **Unclear risk**  Reason: Fear of recurrence was measured with a shortened version of a subscale of the Fear of Cancer Recurrence Inventory. Although the internal consistency within the current sample was high, it is unclear whether this shortened measure is validated. |
| Kepak, T., et al., *Late effects of childhood cancer recorded at a single outpatient clinic over the course of one year: implications for the follow-up care.* Neoplasma, 2022. **69**(4): p. 983-992.  Country: Czech Republic | Fear of infertility  Fear of cancer recurrence  Fear of late effects | Cross-sectional | N: 133  Sex: 45.1% female  Age at study entry: Mean 27.9 SD not reported (range 18-53)  Age at primary cancer diagnosis: Mean 10.4 SD not reported (range 0-18)  Follow-up: Time since diagnosis: Mean 16.8 SD not reported (range 5-43)  Cancer type: Mixed, Malignant lymphomas most common (33.1%)  Treatment type: Chemotherapy (98.5%); Radiotherapy (51.9%); Surgery (42.9%); Hematopoietic stem cell transplantation (11.3%)  Treatment era: diagnosed between 1979 and 2016 | No control group | Cancer-related worries  Selected items from the clinic’s screening questionnaire, which was based on the Czech SF-36, version 1, and supplemented with questions on treatment-related late effects and CCS’ concerns associated with late effects.  Some and major concerns were not specified. | Fear of infertility  No concerns: 32%;  Concerns ^d^: 68% (some: 30%, major: 38%)  Fear of tumor recurrence  No concerns: 20%;  Concerns ^d^: 80% (some: 40%, major: 40%)  Fear of late effects  No concerns: 17%;  Concerns ^d^: 83% (some: 45%, major: 38%) | Not reported | A. Selection bias  **Unclear risk**  Reason: The original cohort of eligible survivors is unknown.  B. Attrition bias  **Low risk**  Reason: At least 132 out of 133 CCS (99.2%) had an outcome assessment on the cancer-specific fears.  C. Confounding  Not applicable, associated factors not analyzed.  D. Measurement bias  **Unclear risk**  Reason: The measure for cancer-related worries is based on the SF-36, and supplemented with additional questions. It is unclear whether the adjusted measure is validated. |
| Langeveld, N.E., et al., *Educational achievement, employment and living situation in long-term young adult survivors of childhood cancer in the Netherlands.* Psychooncology, 2003. **12**(3): p. 213-25.  Country: The Netherlands | Worries about infertility ^a^  Worry about children getting cancer  ^h j^ | Cross-sectional | N: 500  Sex: 47% female  Age at evaluation: Mean 24 SD 5.1 (range 16-49), Median 22  Age at primary cancer diagnosis: Mean 8 SD 4.7 (range 0-19), Median 8  Follow-up: Time since therapy completion: Mean 15 SD 5.8 (range 5-33), Median 15  Cancer type: Mixed, Leukemia most common (21%)  Treatment type: Chemotherapy + radiation (28%); Chemotherapy + radiation + surgery (19%); Chemotherapy + surgery (22%); Radiation + surgery (4%); Single-modality therapy (27%)  Treatment era: diagnosed between 1963 and 1992 | N/type: 1,092 young adults without a cancer history (age and gender matched)  Sex: 55% female  Age at evaluation: Mean 26 SD 5.2 (range 15-53), Median 26 | Worries about infertility and offspring Structured self-reported questionnaire including 1) Worries about infertility (1 item), and 2) Worries about health of their children (1 item). Outcome options not provided. | Worries about infertility:  Total CCS ^c^: 58.6%;  Total controls ^c^: 43.2%;  Male CCS: 53%;  Male controls: 29%;  Female CCS: 65%;  Female controls: 55%  Significantly more worries in CCS than in the control group (p < 0.001), adjusted for sex.  Worries about children getting cancer:  Total CCS  ^c^: 45.2%;  Total controls ^c^: 32.2%;  Male CCS: 40%;  Male controls: 30%;  Female CCS: 51%;  Female controls: 34%  Significantly more worries in CCS than in the control group (p < 0.001), adjusted for sex. | Not reported | A. Selection bias  **Unclear risk**  Reason: The original cohort of eligible survivors is unknown. It should be noted that the authors reported an overrepresentation of leukemia and lymphoma patients in the study group as compared to all patients being alive at the time of the study.  B. Attrition bias  **Low risk**  Reason: The outcome was assessed for 100% of the study group.  C. Confounding  Not applicable, associated factors not analyzed  D. Measurement bias  **High risk**  Reason: Worries about infertility, and worries about health of their children were assessed with one single question that was not validated. |
| Langeveld, N.E., et al., *Quality of life, self-esteem and worries in young adult survivors of childhood cancer.* Psychooncology, 2004. **13**(12): p. 867-81.  Country: The Netherlands | Worry about having a relapse  Worry about having another cancer when older | Cross-sectional | N: 400 CCS attending the long-term follow-up clinic  Sex: 45% female  Age at evaluation: Mean 24.0 SD 4.9, (range 16-49), Median 23  Age at primary cancer diagnosis: Mean 8.0 SD 4.6 (< 19)  Follow-up: Time since treatment completion: Mean 16.0 SD 5.6 (≥ 5)  Cancer type: Leukemia/non-Hodgkin’s lymphoma (48)%; Solid tumor (41)%; Brain/CNS tumor (11%)  Treatment type:  Chemotherapy (with/ without surgery) (42%);  Radiotherapy (with/ without surgery) (9.0%); Combination therapy (chemotherapy and radiotherapy with/ without surgery) (49.0%)  Treatment era: not reported | No control group for cancer-related worries | Cancer-related worries  Assessed with the self-reported Worry questionnaire (18 items) of which 5 items (Cronbach’s α: 0.80) addressed cancer-related worries. The results of 3 items were reported^i^. Responses on a 4-point scale from 1 (never worry) to 4 (worry a lot). | Worry about having a relapse present in 54% of CCS  Worry about having another cancer when older present in 50% of CCS | Not reported | A. Selection bias  **Unclear risk**  Reason: The original cohort of eligible survivors is unknown.  B. Attrition bias  **Low risk**  Reason: There is an assessment on cancer-related worries for all (100%) participants.  C. Confounding  Not applicable, associated factors not analyzed.  D. Measurement bias  **Low risk**  Reason: Cancer-related worries were measured with a validated and reliable questionnaire. |
| Maas, A., et al., *Positive and negative survivor-specific psychosocial consequences of childhood cancer: the DCCSS-LATER 2 psycho-oncology study.* J Cancer Surviv, 2023.  Country: The Netherlands | Sibling concerns  Life challenges: Worries about death, worries about health  Relationship concerns  Worries about infertility  ^a^  Worries about offspring  ^j^ | Cross-sectional | N: 1,713  Sex: 48.9% female  Age at evaluation: Mean 35.97 SD 9.32 (range 18.29-70.88)  Age at primary cancer diagnosis: Mean 6.78 SD 4.73 (range 0.00-17.96)  Follow-up: Time since diagnosis: Mean 29.19 SD 8.49 (range 15.34-55.01)  Cancer type: Mixed, Leukemia most common (34.4%)  Treatment type:  Chemotherapy (87.5%); Surgery (50.4%); Radiotherapy (39.3%)  Treatment era: CCS diagnosed between 1963 and 2001 | No control group | Impact of Cancer – Childhood Cancer (IOC-CS)  Structured self-reported questionnaire about the impact of childhood cancer on various life domains including questions about cancer-related worries:  - Sibling concerns: 1 item about worries;  - Life challenges: 3 items about worries;  - Relationship concerns: 2 items about worries in partnered CCS, 4 items about worries in non-partnered CCS;  - Separate items not represented in scales:  ‘Worry about fertility’, ‘Worry about my children getting cancer’, ’worry about my children’s health’.  Range item scores:  1 (not at all) to 5 (very much). | Mean item scores  Sibling concerns  Worry how cancer affected siblings: Mean 2.11 SD 1.20  Life challenges  Worry about health: Mean 2.19 SD 1.09  Afraid to die: Mean 1.76 SD 1.14  Worry dying at young age: Mean 1.68 SD 1.06  Relationship concerns partnered  Worry partner will leave if cancer returns: Mean 1.31 SD 0.77  Worry about having sex with partner: Mean 1.43 SD 0.94  Relationship concerns non-partnered  Worry about telling potential partner about fertility: Mean 1.98 SD 1.28.  Worry about having no relationship: Mean 2.26 SD 1.27  Worry about having sex: Mean 1.68 SD 1.08  Worry about telling potential partner about cancer: Mean 1.62 SD 1.03  Worry about fertility  Worry about fertility: Mean 1.94 SD 1.32  Worry about offspring  Worry about children getting cancer: Mean 2.01 SD 1.20  Worry about children’s health: Mean 2.07 SD 1.22  Prevalence  Sibling concerns  Worry how cancer affected siblings:  Not at all/a little bit: 65.5%;  Some impact: 19.1%;  Quite a bit/very much: 15.4%  Life challenges  Worry about health:  Not at all/a little bit: 65.3%;  Somewhat: 21.7%;  Quite a bit/very much: 12.9%  Afraid to die:  Not at all/a little bit: 78.6%;  Somewhat: 11.2%;  Quite a bit/very much: 10.1%  Worry dying at young age: Not at all/a little bit: 81.0%;  Somewhat: 11.1%;  Quite a bit/very much: 7.9%  Relationship concerns partnered  Worry partner will leave if cancer returns:  Not at all/a little bit: 91.2;  Somewhat: 5.2%;  Quite a bit/very much: 3.6%  Worry about having sex with partner:  Not at all/a little bit: 88.1%;  Somewhat: 5.9%;  Quite a bit/very much: 6.0%  Relationship concerns non-partnered  Worry about telling potential partner about fertility:  Not at all/a little bit: 71.7%;  Somewhat: 12.1%;  Quite a bit/very much: 16.2%  Worry about having no relationship:  Not at all/a little bit: 59.2%;  Somewhat: 21.4%;  Quite a bit/very much: 19.4%  Worry about having sex:  Not at all/a little bit: 80.1%;  Somewhat: 10.8%;  Quite a bit/very much: 9.1%  Worry about telling potential partner about cancer: Not at all/a little bit: 83.0%;  Somewhat: 8.4%;  Quite a bit/very much: 8.6%  Worry about fertility  Worry about fertility:  Not at all/a little bit: 71.4%;  Somewhat: 12.7%;  Quite a bit/very much: 15.9%  Worry about fertility in age group 18-30:  Not at all/a little bit: 52.0%;  Somewhat: 23.1%;  Quite a bit/very much: 24.9%  Worry about offspring  Worry about children getting cancer:  Not at all/a little bit: 69.3%;  Somewhat: 17.2%;  Quite a bit/very much: 13.5%  Worry about children’s health:  Not at all/a little bit: 68.2%;  Somewhat: 16.9%;  Quite a bit/very much: 14.9% | Not reported on item level | A. Selection bias  **High risk**  Reason: 5,391 CCS were alive and eligible, and 1,713 CCS completed the questionnaires (31.8%).  B. Attrition bias  **Low risk**  Reason: a minimum of 1560 out of 1713 CCS (91.1%) had an assessment on the cancer-related worries.  C. Confounding  Not applicable, associated factors not analyzed.  D. Measurement bias  **Low risk**  Reason: A standardized, validated, and reliable questionnaire was used to measure cancer-related worries. The questionnaire was validated on scale level; items were used in the present study. |
| McDonnell, G.A., et al., *Prevalence and predictors of cancer-related worry and associations with health behaviors in adult survivors of childhood cancer.* Cancer, 2021. **127**(15): p. 2743-2751.  Country: USA | Worry about cancer recurrence  Worry about cancer-related physical problems  Worry about developing another cancer | Cross-sectional | N: 3,211  Sex: 48.6% female  Age at evaluation: Mean 31.2 SD 8.4 (≥ 18)  Age at primary cancer diagnosis: Mean 8.4 SD 5.6 (range not reported, diagnosis in childhood)  Follow-up: Time since diagnosis: Mean 22.8 SD 8.3 (≥ 10)  Cancer type: Mixed, Leukemia most common (35.6%)  Treatment type:  Chemotherapy (85.0%); Surgery (62.4%); Radiotherapy (56.8%)  Treatment era: not reported | No control group | Cancer-related worries  assessed with 3 items: 1) ‘I am worried about my cancer coming back’;2) ‘I am concerned about physical problems related to my cancer’; and 3) ‘I am concerned about developing another cancer’. Range item scores: 1 (strongly disagree) to 5 (strongly agree). Responses were classified as “worried” if participants responded ‘agree’ or ‘strongly agree’.  Psychological and somatic symptoms  Emotional distress was assessed with the anxiety and depression subscales from the Brief Symptom Inventory 18. T scores ≥ 63 represented elevated anxiety/depression.  Pain intensity  Assessed with an item from the 36-Item Short Form Health Survey: ‘How much bodily pain have you had during the past 4 weeks?’. Responses on a 6-point Likert scale from ‘none’ to ‘very severe’. Pain was coded as present when CCS reported moderate to very severe bodily pain.  Pain interference  Assessed with an item from the 36-Item Short Form Health Survey: ‘During the past 4 weeks, how much did pain interfere with your normal work (including both work outside the home and housework)?’. Responses on a 5-point Likert scale from 1 (not at all) to 5 (extremely).  Daily pain interference was coded as present when CCS reported moderate to extreme interference. | Worry about relapse present in 33% of CCS (95% CI: 31.2%- 34.4%)  Worry about cancer-related physical problems present in 45% of CCS (95% CI: 43.5%-46.9%)  Worry about developing another cancer/ second malignant neoplasm (SMN) present in 64% of CCS (95% CI: 62.6%-65.9%)  Worry about both cancer recurrence and developing another cancer present in 31% of CCS (95% CI: 29.4%-32.6%) | Univariable analysis  Females (vs males)  More likely to worry about relapse (36.9% (95% CI: 34.5%-39.3%) vs 29.0% (95% CI: 26.8%-31.2%))  More likely to worry about developing another cancer (70.2% (95% CI: 67.9%-72.5%) vs 58.6% (95% CI: 56.2%-61.0%))  CCS with a history of relapse or SMN (vs without such a history)  More likely to worry about relapse (p: 0.0001);  More likely to worry about physical problems related to cancer (p: 0.0008);  More likely to worry about developing another tumor (p: 0.0007). Prevalence not reported.  Multivariable analysis  Model 1 (Treatment Exposures): surgery, chemotherapy, cranial radiation, non-cranial radiation therapy (CRT), relapse/SMN (all, yes vs no), and covariates age at diagnosis, time since diagnosis, sex, and race).  Significant factors model 1  Worry about relapse  History of non-CRT (yes vs no): RR: 1.14 (95% CI: 1.01-1.29)  Relapse/SMN (yes vs no): RR: 1.18 (95% CI: 1.05-1.32)  Not significant  Surgery, chemotherapy, history of CRT  Worry about developing a SMN:  History of non-CRT (yes vs no): RR: 1.08 (95% CI: 1.02-1.15)  Not significant  Surgery, chemotherapy, history of CRT, relapse/SMN  Worry about physical problems related to cancer:  History of chemotherapy (yes vs no): RR: 1.21 (95% CI: 1.07-1.37)  History of CRT (yes vs no): RR: 1.35 (95% CI: 1.23-1.49)  History of non-CRT (yes vs no): RR: 1.33 (95% CI: 1.20-1.46)  Not significant  Surgery, relapse/SMN  Model 2 (Chronic conditions): obesity (vs underweight/normal), endocrine, musculoskeletal, cardiovascular, sexual reproductive, pulmonary, gastrointestinal, and neurologic (grade 3/4 vs grade 0/1/2), and covariates age at evaluation, sex, race.  Significant factors model 2  Worry about relapse:  Overweight/obese (vs underweight/normal): RR: 1.18 (95% CI: 1.06-1.32)  Neurologic disorder (grade 3/4 vs grade 0/1/2): RR: 1.18 (95% CI: 1.04-1.35)  Not significant  Endocrine disorder, musculoskeletal disorder, cardiovascular disorder, sexual reproductive disorder, pulmonary disorder, gastrointestinal disorder  Worry about SMNs:  Overweight/obese (vs underweight/normal): RR: 1.07 (95% CI: 1.02-1.14)  Cardiovascular disorder (grade 3/4 vs grade 0/1/2): RR: 1.09 (95% CI: 1.02-1.17)  Not significant  Endocrine disorder, musculoskeletal disorder, pulmonary disorder, gastrointestinal disorder, neurologic disorder  Worry about physical problems related to cancer:  Overweight/obese (vs underweight/normal): RR: 1.12 (95% CI: 1.03-1.21)  Cardiovascular disorder (grade 3/4 vs grade 0/1/2): RR: 1.14 (95% CI: 1.04-1.26)  Sexual reproductive disorder (grade 3/4 vs grade 0/1/2): RR: 1.25 (95% CI: 1.15-1.36)  Neurologic disorder (grade 3/4 vs grade 0/1/2): RR: 1.20 (95% CI: 1.09-1.32)  Not significant  Endocrine disorder, musculoskeletal disorder, pulmonary disorder, gastrointestinal disorder  Model 3 (Psychological and somatic symptoms): anxiety (yes vs no), depression (yes vs no), pain intensity (none to moderate to very severe bodily pain vs a little bit of pain), and pain interference (moderate to extreme pain interference vs not at all to mild pain interference), and covariates age at evaluation, sex, race, current health insurance coverage, education level, household income, marital status).  Significant factors model 3  Worry about relapse:  Anxiety (yes vs no): RR: 1.43 (95% CI: 1.22-1.68)  Depression (yes vs no): RR: 1.23 (95% CI: 1.04-1.44)  Pain intensity (none to moderate to very severe bodily pain vs a little bit of pain): RR: 1.20 (95% CI: 1.02-1.41)  Not significant  Pain interference  Worry about SMNs:  Anxiety (yes vs no): RR: 1.17 (95% CI: 1.09-1.26)  Depression (yes vs no): RR: 1.12 (95% CI: 1.04-1.21)  Pain interference (moderate to extreme pain interference vs not at all to mild pain interference): RR: 1.14 (95% CI: 1.05-1.24)  Not significant  Pain intensity  Worry about physical problems related to cancer:  Anxiety (yes vs no): RR: 1.35 (95% CI: 1.21-1.49)  Depression (yes vs no): RR: 1.17 (95% CI: 1.06-1.31)  Pain intensity (none to moderate to very severe bodily pain vs a little bit of pain): RR: 1.38 (95% CI: 1.23-1.54)  Pain interference (moderate to extreme pain interference vs not at all to mild pain interference): RR: 1.16 (95% CI: 1.03-1.30)  Not significant  None | A. Selection bias  **High risk**  Reason: 5,875 CCS were alive and eligible, and 3,211 CCS completed the questionnaires (54.7%).  B. Attrition bias  **Unclear risk**  Reason: it is unclear if all participants have an outcome assessment on cancer-related worries.  C. Confounding  **Low risk for multivariable analysis, high risk for univariable analysis.**  Reason: There is accounted for age at evaluation and sex in the multivariable analysis, but not in the univariable analysis. In Model 1, there was adjusted for age at diagnosis and time since diagnosis, and thus indirectly for age at evaluation.  D. Measurement bias  **High risk**  Reason: cancer-related worries were assessed with 3 single items that were not standardized or validated. |
| van Erp, L.M.E., et al., *A vulnerable age group: the impact of cancer on the psychosocial well-being of young adult childhood cancer survivors.* Support Care Cancer, 2021. **29**(8): p. 4751-4761.  Country: The Netherlands | Sibling concerns  Life challenges: worries about death, worries about health  Relationship concerns | Cross-sectional | N: 151  Sex: 61.6% female  Age at evaluation: Mean 24.1 SD 3.6 (range 18-30)  Age at primary cancer diagnosis: Mean 10.5 SD 4.5 (range 0.4-17)  Follow-up: Time since diagnosis: Mean 13.6 SD 3.8 (range 6-27)  Cancer type: Hematologic cancers 66.9%; CNS tumors 8.6%; Solid tumors 24.5%  Treatment type:  Surgery (61.6%); Chemotherapy (95.4%); Radiotherapy (37.1%); SCT/BMT (7.3%)  Treatment era: not reported | No control group for cancer-related worries | Impact of Cancer – Childhood Cancer (IOC-CS)  Structured self-reported questionnaire about the impact of childhood cancer on various life domains including items about cancer-related worries:  - Sibling concerns: 1 item about worries;  - Life challenges: 3 items about worries;  - Relationship concerns: 2 items about worries in partnered CCS, 4 items about worries in non-partnered CCS  Range item scores:  1 (not at all) to 5 (very much) | Mean item scores  Sibling concerns  Worry how cancer affected siblings: Mean 2.8 SD 1.4  Life challenges  Worry about health: Mean 2.4 SD 1.3;  Afraid to die: Mean 2.1 SD 1.4;  Worry dying at young age: Mean 2.0 SD 1.3  Relationship concerns partnered  Worry partner will leave if cancer returns: Mean 1.7 SD 1.2;  Worry about having sex with partner: Mean 1.4 SD 0.7  Relationship concerns non-partnered  Worry about telling potential partner about fertility: Mean 2.2 SD 1.3;  Worry about having no relationship: Mean 2.2 SD 1.2;  Worry about having sex: Mean 1.8 SD 1.2;  Worry about telling potential partner about cancer: Mean 1.6 SD 0.9 | Not reported | A. Selection bias  **Unclear risk**  Reason: The original cohort of eligible survivors is unknown.  B. Attrition bias  **Low risk**  Reason: There is an outcome assessment on cancer-related worries on at least 142 out of 151 CCS (94.0%).  C. Confounding  Not applicable, associated factors not analyzed.  D. Measurement bias  **Low risk**  Reason: A standardized, validated and reliable questionnaire was used to measure cancer-related concerns. The questionnaire was validated on scale level; items were used in the present study. |

Abbreviations: CCS=childhood cancer survivors, SD=standard deviation, RR=Risk Ratio, OR=Odds Ratio, SCT=Stem Cell Transplantation, BMT=Bone Marrow Transplantation, SMN=Second Malignant Neoplasm, CRT=Cranial Radiation Therapy, RB=retinoblastoma, Gy=Gray

^a^ For the worry about infertility, there is potential overlap in CCS between the study by Langeveld et al. (2003) and the study by Maas et al. (2023).

^b^ This study also reports longitudinal results with lack of concern as an outcome. As this is not the focus of this systematic review, those results are not reported.

^C^ The total prevalence percentage was calculated by weighting the prevalence rates for men and women according to their respective proportions in the sample.

^d^ The total prevalence was calculated by combining the percentages of all relevant subcategories, e.g., some and major concerns.

^e^ The percentage of 56.8% was shown in a figure in the article, while 45.8% was written in the text. Since 56.8% allows the percentages to sum to 100%, we assume the 45.8% in the text to be a typographical error.

^f^ Although 53.1% was reported in the article, we believe this to be a typographical error based on the absolute numbers provided and the fact that 63.1% allows the percentages to sum to 100%.

^g^ Although 25.7% was reported in the article, we believe this to be a typographical error based on the absolute numbers provided and the fact that 27.5% allows the percentages to sum to 100%.

^h^ Worry about children getting cancer was reported by both Langeveld (2003) and Langeveld (2004). However, due to the overlap in study populations, only the results from Langeveld (2003) are presented, as this study included the larger sample size.

^i^ The worry about children getting cancer was described in this study but was not included in this review due to the overlap with Langeveld (2003)

^j^ For the worry about children getting cancer, there is potential overlap in CCS between the study by Langeveld et al. (2003) and the study by Maas et al. (2023).
